# Separation and Characterization of Heterogeneity Among Various Sizes of Outer Membrane Vesicles Derived from the Probiotic *Escherichia coli* Nissle 1917

**DOI:** 10.3390/membranes15050141

**Published:** 2025-05-05

**Authors:** Ning Li, Hongbo Xin, Keyu Deng

**Affiliations:** The National Engineering Research Center for Bioengineering Drugs and the Technologies, Jiangxi Province Key Laboratory of Bioengineering Drugs, Institute of Translational Medicine, Jiangxi Medical College, Nanchang University, Nanchang 330031, China; xinhb@ncu.edu.cn

**Keywords:** outer membrane vesicles, different sizes, membrane filtration, *Escherichia coli* Nissle 1917, heterogeneity, macrophage

## Abstract

Outer membrane vesicles (OMVs) are extracellular vesicles secreted by Gram-negative bacteria with diameters of 20–250 nm. OMVs contain various biologically active substances from their parent bacteria, such as proteins, lipids, and nucleic acids. *Escherichia coli* Nissle 1917 (EcN) is a Gram-negative probiotic that resides in the human intestine. EcN-derived OMVs are pivotal in modulating intestinal immune responses. However, few studies have addressed the heterogeneity of EcN-derived OMVs in terms of size, significantly limiting the research on their clinical applications. Currently, there are a lack of feasible methods for obtaining EcN-derived OMVs of different sizes. To address this knowledge gap, we developed a membrane filtration method to isolate EcN-derived OMVs of varying sizes. In this study, we first used gradient filtration to isolate high-purity EcN-derived OMVs and conducted a proteomic analysis. Subsequently, we used membrane filtration to separate the EcN-derived OMVs by size. We successfully obtained EcN-derived OMVs of three specific sizes: <50 nm, 50–100 nm, and 100–300 nm. We then performed proteomic analyses of these EcN-derived OMVs and compared their protein profiles. Finally, we compared the ability of each EcN-derived OMV type to induce RAW264.7 macrophages to secrete the pro-inflammatory factor interleukin (IL)-1β and the anti-inflammatory factor IL-10. The EcN-derived OMVs contained 646 different proteins overall; those of different sizes contained different protein types. Among them, the EcN-derived OMVs in the <50 nm group contained significantly fewer proteins (262 different types in total) than those in the 50–100 nm (1603 types) and 100–300 nm (1568 types) groups. Furthermore, the <50 nm group had fewer membrane proteins (40) than the 50–100 nm (215) and 100–300 nm (209) groups. We also found that RAW264.7 macrophages secreted different concentrations of IL-1β and IL-10 following co-incubation with the three EcN-derived OMV types. The 50–100 nm EcN-derived OMV group showed a stronger effect in terms of inducing inflammatory cytokine secretion compared to the other two groups. This study provides direct experimental evidence that EcN-derived OMVs of different sizes exhibit heterogeneous properties.

## 1. Introduction

Bacterial outer membrane vesicles (OMVs) are extracellular vesicles with defined membrane structures that are actively secreted by Gram-negative bacteria [1]. Their particle size distribution is approximately 20–250 nm, and they cannot replicate [2]. OMVs contain various parent bacterial components that endow them with unique biological functions, including lipopolysaccharides, peptidoglycans, phospholipids, proteins, and nucleic acids (e.g., DNA and RNA) [3,4,5,6]. OMVs are crucial mediators of intercellular communication in bacteria–bacteria and bacteria–host interactions [7,8,9,10]. OMVs regulate various bacterial interactions, including biofilm formation, antibiotic resistance, and the killing of competing bacteria [11]. OMVs also stimulate the activities of epithelial cells, endothelial cells, and various immune cells in the host [12,13]. Proteomic analyses of OMVs provide information on their protein composition, biogenesis, and function. The gradual development of liquid chromatography with tandem mass spectrometry (LC–MS/MS) over recent years has played a pivotal role in promoting the development of proteomics research into OMVs [14,15,16]. Lee et al. used LC–MS/MS to demonstrate that OMVs derived from *Escherichia coli* bacteria contain various outer membrane proteins and certain porins, such as OmpA, OmpF, and NmpC. These proteins have immunostimulatory activities and can induce leukocyte migration [17]. Kwon et al. reported that OMVs derived from *Acinetobacter baumannii* contained 132 proteins, of which 43 are cytoplasmic, 26 are outer membrane proteins, 8 are inner membrane proteins, 6 are periplasmic, and the rest have an unknown localization [18]. Choi et al. reported that OMVs derived from *Pseudomonas aeruginosa* contained 338 proteins [19]. Among these proteins, the outer membrane proteins are mainly involved in the assembly of bacterial flagella, pore formation, the transport of specific substrates, and outer-membrane stability. The other proteins were found to be related to neutrophil-mediated killing.

The intestine is the largest immune organ in the body. The intestinal mucosal innate immune system, which comprises intestinal mucosal tissue, immune cells, and immune molecules, represents the body’s primary line of defense against pathogenic invasion. Upon interaction with pathogenic microorganisms, the intestinal mucosal innate immune system can elicit an immune response, thereby contributing to the body’s defense mechanisms [20,21]. As important innate immune cells, macrophages are among the immune cell models commonly used to study innate immune responses in vitro, and they play crucial roles in intestinal mucosal immunity. *Escherichia coli* Nissle 1917 (EcN) is a Gram-negative probiotic that resides in the human intestine. The research has indicated that EcN positively contributes to regulating host immune function and treating intestinal diseases [22]. Compared to EcN probiotics, EcN-derived OMVs exhibit a high propensity to disseminate, traverse the mucus layer, and migrate directly to the intestinal submucosal tissue to interact with host immune-system cells. Importantly, EcN-derived OMVs preserve the beneficial and immunogenic characteristics of the parent bacterium while mitigating the potential risks associated with administering live bacteria to the host. Canas et al. reported that EcN-derived OMVs mediated intestinal anti-inflammatory responses and barrier-protective effects in a mouse model of DSS-induced intestinal inflammation [23]. Fábrega et al. demonstrated that the oral administration of EcN-derived OMVs in a mouse model effectively reduced pro-inflammatory factor levels, increased the expression of intestinal barrier markers, and consequently inhibited the progression of DSS-induced colitis [24]. Alvarez et al. reported that EcN-derived OMVs activated intestinal mucosal immune and defense responses. Furthermore, these EcN-derived OMVs enhance barrier function by increasing the expression of tight junction proteins in intestinal epithelial cells [25]. There have been few studies on the heterogeneity of EcN-derived OMVs in terms of size. Isolating EcN-derived OMVs of different sizes represents the first and most important step toward conducting research regarding their heterogeneity. Currently, fast, effective, and efficient methods for the refined preparation of OMVs of different sizes are lacking. Ultrafiltration is currently used as a method for the isolation of OMVs [2]. It separates them based on their distinct physical characteristics using a combination of filter membranes of different sizes to facilitate the separation process. Inspired by the principles of ultrafiltration, we developed a membrane filtration method that is convenient and effective for separating EcN-derived OMVs of different sizes. This method has the advantages of not requiring any additional reagents, being simple to execute, being low-cost, being able to preserve the biological activity of OMVs, and being scalable for industrial mass production.

EcN-derived OMVs are pivotal in modulating intestinal immune responses. Compared to the probiotic EcN itself, EcN-derived OMVs offer unique advantages [26]. There are relatively few studies addressing the heterogeneity of EcN-derived OMVs of varying sizes, which significantly restricts research into their clinical applications. In this study, we first used the gradient filtration method to isolate high-purity OMVs derived from EcN and then conducted proteomic analyses on the isolated EcN-derived OMVs. Second, we used the filtration method to obtain EcN-derived OMVs of three specific sizes: <50 nm, 50–100 nm, and 100–300 nm. We then conducted proteomic analyses on the EcN-derived OMVs of three specific sizes. Finally, we analyzed the interactions of EcN-derived OMVs of three specific sizes with RAW264.7 macrophages to characterize their differential effects. Our results showed that the isolated EcN-derived OMVs contained a total of 646 different proteins, and the EcN-derived OMVs of different sizes contained different protein types. Among them, we found that the 50–100 nm group contained the widest variety of proteins compared to those in the <50 nm and 100–300 nm groups. We also found that RAW264.7 macrophages secreted different amounts of IL-1β and IL-10 following co-incubation with the three EcN-derived OMV types. The 50–100 nm EcN-derived OMV group showed a stronger effect in terms of inducing inflammatory cytokine secretion than the other two groups. These findings confirmed that EcN-derived OMVs of varying sizes exhibited heterogeneous characteristics.

## 2. Materials and Methods

### 2.1. RAW264.7 Macrophage Culture

RAW264.7 macrophages were purchased from the Shanghai Cell Biology Institute of the Chinese Academy of Sciences (Shanghai, China). Fetal bovine serum (FBS) and Dulbecco’s Modified Eagle Medium (DMEM) were purchased from Thermo Fisher Scientific (Waltham, MA, USA). Penicillin-streptomycin solution was purchased from Beyotime Biotechnology (Shanghai, China). RAW264.7 macrophages were cultured in DMEM with 10% FBS and 1% penicillin-streptomycin. The cells were cultured in a CO_2_ incubator with a humidified atmosphere of 5% CO_2_/95% air at 37 °C.

### 2.2. Isolation of EcN-Derived OMVs and Size-Based Sorting

#### 2.2.1. Isolation of EcN-Derived OMVs Through Gradient Filtration

The EcN bacterial stock was purchased from Baosai Biological Company (Hangzhou, China). EcN-derived OMVs were obtained via the gradient filtration protocol previously reported by our research group [27]. We optimized the protocol for the gradient filtration and significantly improved the purity of the OMV samples by repeating the pretreatment and washing steps multiple times. The detailed protocol for the isolation of EcN-derived OMVs is described below. Aliquots of EcN glycerol cryopreservation solution (100 µL) were inoculated into 10 mL of sterile LB culture medium (Sevier Biotechnology, Wuhan, China) at a 1:100 ratio and cultured at 37 °C at 200 rpm for 8 h. Subsequently, 200 µL of this seed mixture was inoculated into 200 mL of sterile LB culture medium and cultured at 37 °C at 200 rpm for 18 h. Finally, 200 mL of EcN culture medium was collected. The EcN culture medium was then centrifuged at 15,000× *g* at 4 °C to remove the bacterial cells and any sediments. The supernatant was subsequently filtered through a 0.45 µm filter (Millipore, Darmstadt, Germany) to remove residual cellular debris. The resulting supernatant was then filtered through a 0.3 µm filter membrane (Millipore). Finally, the collected filtrate was filtered and centrifuged in an ultrafiltration centrifuge tube (100 kDa, UFC9100; Millipore) at 5000× *g* until the remaining volume was approximately 0.5 mL. Residual proteins were removed via three consecutive PBS washes (12 mL of PBS solution per wash). The ultrafiltration centrifuge tube was then centrifuged at 5000× *g* for 30 min. The final precipitate (i.e., purified EcN-derived OMVs) was dissolved in 200 µL of PBS and stored at –80 °C for later use.

#### 2.2.2. Size-Based Sorting of EcN-Derived OMVs of Varying Sizes

The EcN-derived OMV concentrate was first separated via membrane filtration with different pore sizes (100 nm, UFC30VV25; Millipore) at 5000× *g* for 5 min, with both upper solutions and lower filtrates collected to obtain <100 nm and 100–300 nm concentrates. The <100 nm concentrate was then passed through a 50 nm filter (50 nm, VMWP02500; Millipore) to separate the OMVs into <50 nm and 50–100 nm concentrates. The different OMV classes were then concentrated via ultrafiltration (100 kDa; Millipore) at 5000× *g* for 15 min.

### 2.3. TEM of EcN-Derived OMVs

TEM images were acquired using a JEM1011 electron microscope (JEOL, Tokyo, Japan). The EcN-derived OMV samples were loaded onto TEM copper grids and dried for 20 min. The samples were negatively stained with 2% phosphotungstic acid for 10 min before TEM imaging, which was performed at 80 kV.

### 2.4. NTA of EcN-Derived OMVs

NTA was performed on a NanoSight NS300 instrument (Malvern, Worchestershire, UK). The EcN-derived OMV samples were warmed to room temperature and vortexed before being serially diluted to break apart any aggregates. A 10 µL volume of the resulting mixture was then diluted with PBS to achieve a concentration ranging from 1 × 10^7^ to 1 × 10^9^ particles/mL. The size distribution of the OMVs was then determined via a ZetaView PMX 110 particle matrix at 405 nm.

### 2.5. DLS of EcN-Derived OMVs of Varying Sizes

DLS was performed on a Nano ZS90 instrument (Malvern). The size distribution of the EcN-derived OMVs was characterized via DLS. A 10 µL volume of the resulting mixture was diluted with 1 mL of PBS and added to a DLS test cup to perform the determination.

### 2.6. BCA Protein Quantification of EcN-Derived OMVs

A bicinchoninic acid (BCA) protein assay kit (Beyotime Biotechnology, Shanghai, China) was used to prepare a standard curve per the manufacturer’s instructions. A fixed volume of each EcN-derived OMV sample was then mixed with radioimmunoprecipitation assay lysis solution. The mixture was oscillated at room temperature for 15 min. A 2 µL volume of each EcN-derived OMV sample lysate was obtained, and its absorbance (562 nm) was measured via a SpectraMax M5 full-wavelength microplate reader (Molecular Devices, Sunnyvale, CA, USA). The concentration of the EcN-derived OMVs was then determined according to the BCA standard curve and sample dilution factor.

### 2.7. Purification of EcN-Derived OMV Samples via Size-Exclusion Chromatography

Size-exclusion chromatography (SEC) was performed using the Exosupur^®^ Purification Kit (Beijing Enzekantai Company, Beijing, China, ES914) according to the manufacturer’s instructions: first, the Exosupur^®^ exclusion column was removed from the refrigerator, maintained at 4 °C, and stabilized in an upright orientation using a test tube rack. The column was then allowed to equilibrate to ambient temperature for 30 min. Subsequently, the column was rinsed with 20 mL of PBS buffer, which was twice the column’s volume. After the PBS was completely drained, 1 mL of the sample was added (if the sample was less than 1 mL, PBS was added until the total volume was 1 mL). After all samples were transferred into the sieve plate, 10 mL of the PBS eluent was added. Starting from the first drop of effluent, the effluent was collected using 1.5 mL centrifuge tubes. Each 500 µL of the effluent was marked as one fraction. Next, the OMVs were collected. It was recommended to discard fractions 1–4 and only retain fractions 5–7, as these fractions contained the highest concentration of OMVs. These collected fractions were combined into a single solution. The protein impurities predominantly appeared in the fractions collected after fraction 10, so fractions 10–15 were also collected and combined separately as a mixed protein sample. The collected OMVs and mixed protein solutions were concentrated via ultrafiltration centrifuge tube (100 kDa, UFC9100; Millipore). Subsequently, 200 µL of PBS was added to obtain an OMV concentrate and impure protein concentrate. These concentrates were subjected to quantitative analysis using the BCA protein quantification method.

### 2.8. SDS-PAGE of EcN-Derived OMVs

The SDS-PAGE gel preparation kit was purchased from Beyotime Biotechnology (Shanghai, China). The EcN-derived OMVs (10 µg) were boiled in 5× SDS-PAGE loading buffer for 10 min and then centrifuged (12,000× *g*), and the resulting supernatants were analyzed via SDS-PAGE on a 12% separating gel stained with G-250 protein-staining reagent (Baiolaibo Technology, Beijing, China).

### 2.9. Proteomic Study of EcN-Derived OMVs

Protein expression was verified using Wayen Biotechnology (Shanghai, China) via LC-MS/MS using label-free proteomic analysis, according to their established protocols [28]. Proteomic experiments with EcN-derived OMV samples were performed in biological quadruplicate. Proteomic experiments with EcN-derived OMVs of three specific sizes were performed once each. The LC-MS-MS/MS protocol is described below.

#### 2.9.1. Preprocessing of Proteomic Samples

Equal volumes of protein lysis solution [7 M urea (Sigma, St. Louis, MO, USA), 2% SDS (Sigma), and 1% protease inhibitor cocktail (Thermo Scientific, San Jose, CA, USA)] were added to the EcN-derived OMV samples, after which an ultrasonic cell disrupter (Xinzhi Biotechnology, Ningbo, China) was used for lysis. The samples were repeatedly sonicated for 2 s, with an intervening pause of 5 s, for a total of 1 min, before being lysed on ice for 2 h. The lysed product was then centrifuged at 13,000 rpm for 20 min at 4 °C. The supernatant was collected, and its concentration was measured via BCA quantification (Beyotime Biotechnology, Shanghai, China). The samples (50 µL) of this protein mixture were then added to 10 mM dithiothreitol (DTT; Sigma) and incubated at 37 °C for 1 h. The solutions were then centrifuged at 13,000 rpm for 1 h at 4 °C to collect precipitates. The collected precipitates were added to 1 mL aliquots of 100% acetone (Thermo Scientific) for purification. The samples were then centrifuged at 13,000 rpm for 30 min, the supernatant was discarded, and the precipitate was subjected to repeated washing. The precipitate was then dried at room temperature for 10 min, dissolved in PBS, and incubated with trypsin (Promega, Sunnyvale, CA, USA) at 37 °C overnight for enzymatic digestion. Following centrifugation for 30 min at 13,000 rpm, the peptide precipitate was obtained and desalted via a C18 desalting column (GL Sciences, Tokyo, Japan).

#### 2.9.2. LC-MS/MS Analysis

The drained peptide sample was redissolved in the mobile phase and centrifuged at 20,000× *g* for 10 min, after which the supernatant was collected for injection-processing. The peptide sample was first separated via a nanoElute UPLC liquid-phase system (Brucker, Karlsruhe, Germany). Mobile phase A consisted of 0.1% formic acid (Aladdin, Los Angeles, CA, USA) in water, and the flow rate was 300 μL/min. Mobile phase B [0.1% formic acid (Aladdin) in acetonitrile (Thermo Scientific)] was run at 2% for the first 5 min, ramped to 22% over 45 min, and ramped to 35% over 50 min, before being rapidly increased to 80% for 55 min. The peptide fragments separated by the liquid phase were ionized via the CaptiveSpray source before being input into the timsTOF Pro 2 tandem mass spectrometer (Brucker) for mode detection. The main parameters used in the mass spectrometer were as follows: ion source voltage, 1.5 kV; ion mobility range, 0.76–1.29 V·S/cm^2^; first-level mass spectrometry scanning range, 452–1152 m/z; and peak intensity detected only at >2500. The 452–1152 m/z range was divided into four steps. Each step was divided into seven windows, and a total of 56 windows were used for continuous window fragmentation and information collection. The fragmentation mode was the charge injection device, and the fragmentation energy was 20–59 eV. The mass width of each window was 25. The cycle time of each DIA scan was 1.59 s.

#### 2.9.3. Database Searching, Protein Identification, and Statistical Analyses

The obtained MS/MS data were analyzed via Spectronaut 17 (Biognosys, Schlieren, Switzerland). Database searches were performed via the UniProt database according to a previously reported protocol [29]. For proteins derived from the UniProt database, the UniProt ID was first used to match the GO ID, and the corresponding protein information was retrieved from the UniProt-GOA database on the basis of the GO ID. The following search parameters were used: enzyme digestion of Trypsin, with a parent tolerance of 15 ppm; fragment tolerance of 0.5 Da; minimum peptide length of 5; false discovery rate of 1% at the peptide and protein levels; cysteine carbamidomethylation as fixed modification; and methionine oxidation and protein N-terminal acetylation as variable modifications. The identified proteins were classified according to their subcellular localization and molecular functions via CELLO (http://cello.life.nctu.edu.tw/ (accessed on 1 September 2024)) and InterProScan (http://www.ebi.ac.uk/interpro/ (accessed on 1 September 2024)), respectively [30]. The COG protein functional classification method was used to classify the protein molecules involved in various biological processes.

### 2.10. Internalization of EcN-Derived OMVs of Diverse Sizes by RAW264.7 Macrophages

For the negative control group, 500 µL of PBS was combined with 0.5 µL of DiO membrane dye. In the experimental groups, 20 µg of the EcN-derived OMV samples was diluted in 500 µL of PBS and mixed with 0.5 µL of DiO membrane dye. All reaction mixtures underwent purification using an ultrafiltration centrifuge tube (100kDa) at 6000 rpm for 30 min to remove any unbound dye; two purification cycles were performed for each sample. Then, the precipitate was resuspended in 200 µL of PBS. RAW264.7 macrophages were co-cultured with the purified DiO-labeled EcN-derived OMVs of varying sizes for 16 h. The RAW264.7 macrophages were washed three times with PBS, followed by nuclear staining with Hoechst 33342 for 5 min. After washing with PBS, the RAW264.7 macrophages were added to the cell culture medium for fluorescence imaging.

### 2.11. Effects of EcN-Derived OMVs of Varying Sizes on Inflammatory Cytokine Secretion by RAW264.7 Macrophages

RAW264.7 macrophages were seeded into a 24-well plate at a concentration of 1 × 10^5^/well. The cells were cultured at 37 °C and 5% CO_2_ until 70–80% confluence. A 0.95 mL aliquot of culture medium was added to each well to replace the extracted culture medium, and 50 µL aliquots of EcN-derived OMVs of different sizes at standardized concentrations of 80 µg/mL were added to final concentrations of 4 µg/mL in each well. After the RAW264.7 cells were cocultured with different sizes of EcN-derived OMVs for 24 h, the culture media were collected and centrifuged at 1500× *g* for 10 min at 4 °C, and the supernatants were collected. ELISA kits (Sevier Biotechnology, Wuhan, China) were used to measure the concentrations of IL-1β and IL-10 according to the manufacturer’s instructions.

### 2.12. Statistical Analysis

Statistical analyses were performed via one-way ANOVA. All the data are presented as the mean ± SDs of three independent experiments. Significance was assessed at *p* < 0.05 (*), *p* < 0.01 (**), and *p* < 0.001 (***). All the statistical analyses were performed via GraphPad Prism software 5.0 (San Diego, CA, USA).

## 3. Results

### 3.1. EcN-Derived OMV Isolation via Gradient Filtration

**Figure 1:**
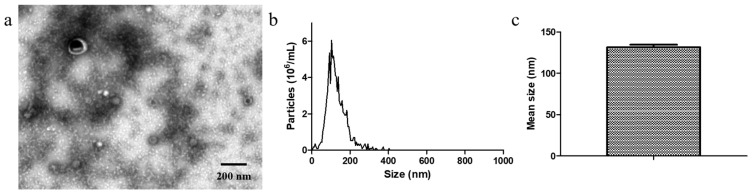
Transmission electron microscopy (TEM) and nanoparticle tracking analysis (NTA) of EcN-derived OMVs. (**a**) TEM images of EcN-derived OMVs (scale bar: 200 nm); (**b**) particle size distribution of EcN-derived OMVs measured via NTA; (**c**) mean size of EcN-derived OMVs. The data are shown as the mean ± SD; n = 3. The mean size refers to the average size of the EcN-derived OMVs. The mean size values were automatically generated in the NTA report.

We used the gradient filtration method that was previously published by our research group to separate OMVs from probiotic EcN bacteria [27]. First, the morphological characteristics of the isolated EcN-derived OMVs were analyzed via transmission electron microscopy (TEM), and their size distributions were characterized via nanoparticle tracking analysis (NTA). The morphologies of the EcN-derived OMVs were clearly observable via TEM (Figure 1a). The TEM results revealed that the isolated EcN-derived OMVs exhibited membrane structures. The NTA results revealed that the mean size of the isolated EcN-derived OMVs was 131.4 ± 3.16 nm, with a size distribution ranging from 20 to 300 nm (Figure 1b,c). These results were consistent with the previously reported structural characteristics and particle sizes of EcN-derived OMVs [31,32], confirming that the isolation procedure was successful. Next, to investigate the potential presence of protein contamination in the EcN-derived OMV samples, we used size-exclusion chromatography (SEC) to purify the samples. SEC is currently used as an effective method for achieving high-purity OMVs [2]. Our experimental results demonstrated that although we successfully acquired a concentrate of EcN-derived OMVs purified through SEC, we did not isolate a concentrated solution of the contaminating proteins. Importantly, to further confirm the purity of the EcN-derived OMVs obtained through gradient filtration, a protein analysis of the EcN-derived OMVs obtained using the two methods (SEC and gradient filtration) was carried out with SDS-PAGE. The experimental results, presented in Figure S1, demonstrated that the protein bands identified in the EcN-derived OMVs obtained using the gradient filtration corresponded to those observed via SEC. These results suggest that the purity of the EcN-derived OMVs obtained via gradient filtration is comparable to that achieved with SEC. Finally, in order to explore the therapeutic effect of EcN-derived OMVs on macrophages, we assessed the secretion of the pro-inflammatory factor IL-1β and anti-inflammatory factor IL-10 by RAW264.7 cells treated with the EcN-derived OMVs purified via SEC. As illustrated in Figure S2, we found that the RAW264.7 macrophages exhibited significantly elevated levels of IL-1β and IL-10 compared to the PBS control group (0 µg/mL of EcN-derived OMVs). Our results are consistent with those of a related study that reported that EcN-derived OMVs have the capacity to induce pro-inflammatory and anti-inflammatory responses in RAW264.7 macrophages [33]. In conclusion, the above results indicated that we efficiently isolated high-purity EcN-derived OMVs via gradient filtration.

### 3.2. Proteomic Analysis of EcN-Derived OMVs

**Figure 2:**
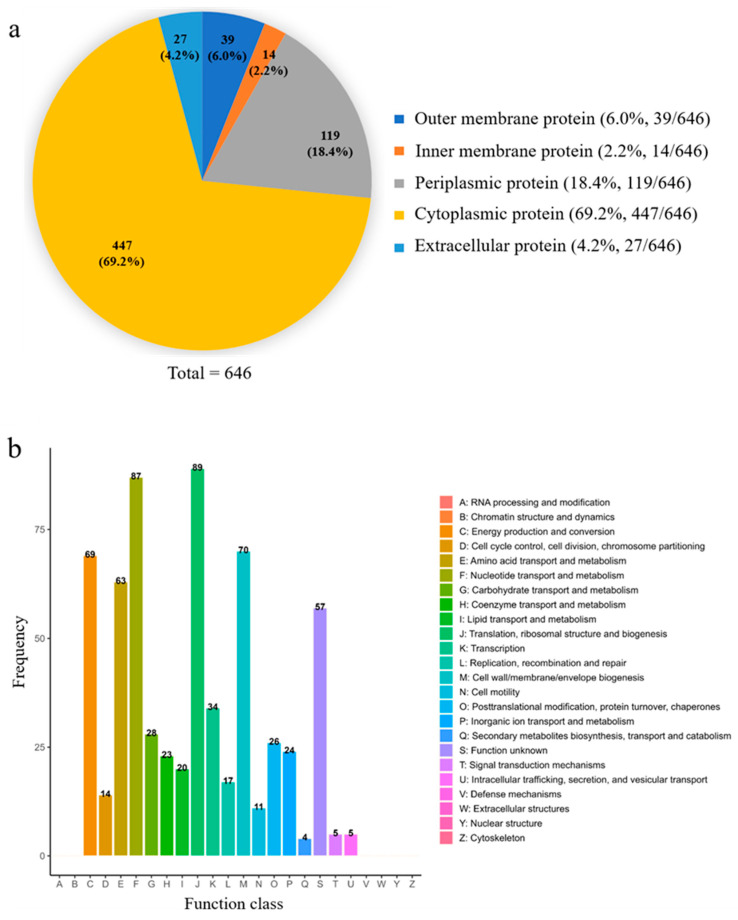
Proteomic analysis of EcN-derived OMVs. (**a**) Subcellular locations of the 646 common proteins identified via proteomic analysis. (**b**) We identified 646 common proteins according to a functional classification performed via the Clusters of Orthologous Groups (COG) of proteins database. The horizontal axis represents the classification content, and the vertical axis represents the relative content of the corresponding number of functional proteins.

We first conducted a protein analysis of the isolated EcN-derived OMVs via sodium dodecyl sulfate-polyacrylamide gel electrophoresis (SDS-PAGE) obtained from three groups of repeated experiments (Figure S3). The SDS-PAGE results revealed the abundance of proteins in the isolated EcN-derived OMVs. Importantly, in comparison to the SDS-PAGE experimental results for EcN-derived OMVs reported in the literature, the protein bands in this study were relatively singular, exhibiting a minimal presence of contaminating protein bands [26]. Furthermore, the bands of the outer membrane marker protein OmpA were prominently visible. These results further demonstrate that the EcN-derived OMVs we obtained were highly pure and exhibited no contamination from other proteins. Notably, the distribution of protein bands across the three experimental groups was consistent, indicating that the gradient filtration method used to separate EcN-derived OMVs exhibited high repeatability. Next, we performed LC-MS/MS on the isolated EcN-derived OMVs from four groups of repeated experiments, identifying 1003 proteins (Figure S4). Among these, 646 common proteins (64.4%, 646/1003) were consistently identified across all four groups. According to the subcellular locations of the 646 common identified proteins within the parent bacteria (Figure 2a and Table S1), the common proteins were classified into the following five groups: cytoplasmic (69.2%, 447/646), outer membrane (6.0%, 39/646), periplasmic (18.4%, 119/646), inner membrane (2.2%, 14/646), and extracellular (4.2%, 27/646). The subcellular locations and clusters of orthologous group (COG) functions of the 646 common proteins are summarized in Table S1. Importantly, our proteomic analysis revealed that the enriched EcN-derived OMV samples, as previously reported in the literature, contained characteristic proteins that could regulate host immune responses and provide protection against diseases. These characteristic proteins included notable outer membrane proteins (such as OmpA, OmpF, and OmpC) [33]. According to the COG functions of protein classifications (Figure 2b and Table S1), most of these common proteins were related to the biological processes of ribosomal structure/biogenesis (n = 89), the transportation and metabolism of nucleotides (n = 87), cell wall/membrane biogenesis (n = 70) and energy production and conversion (n = 69). Some proteins were also involved in transcription (n = 34); posttranslational modification and protein turnover (n = 26); replication and repair (n = 17); cycle control and cell division (n = 14); cell motility (n = 11); and the transportation and metabolism of carbohydrates (n = 28), inorganic ions (n = 24), coenzymes (n = 23), and lipids (n = 20). A few proteins related to signal transduction (n = 5), vesicular transport (n = 5), and secondary metabolite biosynthesis (n = 4) were also identified. Thus, EcN-derived OMVs contained various proteins that potentially mediated their biological functions.

### 3.3. Segmentation of EcN-Derived OMVs of Varying Sizes and Proteomic Analysis

The primary consideration in studying the heterogeneity of differently sized EcN-derived OMVs is how to obtain samples that meet the requirements. Currently, feasible methods for obtaining EcN-derived OMVs of different sizes are lacking. Liu et al. isolated extracellular vesicles of different sizes from cultured media with 22Rv1 prostate cancer cells via ExoTIC devices with two key pore sizes (30 and 50 nm) [34]. On the basis of this precedent, we developed a membrane filtration method to achieve the segmented separation of EcN-derived OMVs. A schematic detailing the membrane filtration method for separating OMVs of different sizes is presented in Figure S5. We used two membranes with different pore sizes (50 and 100 nm) to successfully divide the high-purity isolated EcN-derived OMVs into three distinct size groups: <50 nm, 50–100 nm, and 100–300 nm.

**Figure 3:**
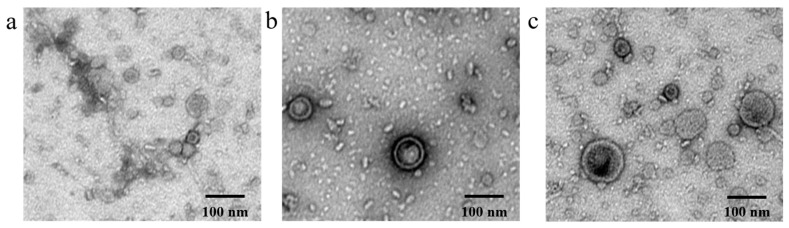
TEM images of EcN-derived OMVs of three specific sizes (<50 nm, 50–100 nm, and 100–300 nm): (**a**) <50 nm group; (**b**) 50–100 nm group; (**c**) 100–300 nm group. Scale bar: 100 nm.

The procedure did not require the use of exogenous substances; therefore, the EcN-derived OMVs of three specific sizes were considered highly pure and free from contamination from other proteins. First, we conducted a TEM analysis of EcN-derived OMVs of three specific sizes. The TEM results clearly revealed the morphology of these EcN-derived OMVs (Figure 3). Notably, TEM results indicated that the three groups of EcN-derived OMVs displayed distinct size variations. Second, we used dynamic light scattering (DLS) to measure the particle size distributions of the three EcN-derived OMV classes (Figure S6). The particle size distributions of the three EcN-derived OMV classes were consistent with their respective size distribution characteristics. In conclusion, by using the membrane filtration method, we successfully obtained EcN-derived OMVs of three specific sizes: <50 nm, 50–100 nm, and 100–300 nm.

**Figure 4:**
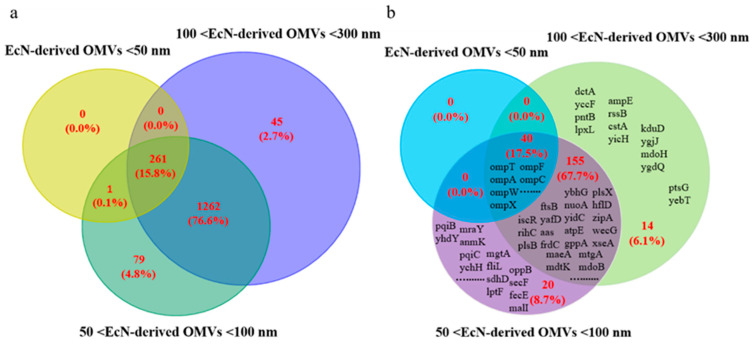
Proteomic analysis of the three EcN-derived OMV groups. (**a**) Venn diagram showing the unique and common proteins among the three groups of EcN-derived OMVs. (**b**) Venn diagram showing the unique and common membrane proteins among the three groups of EcN-derived OMVs.

We used LC-MS/MS analysis to perform proteomic analyses of the three groups of EcN-derived OMVs. As shown in Figure 4a and Table S2, we identified a total of 1648 unique proteins in the <50 nm, 50–100 nm, and 100–300 nm groups, comprising 15.8% (262/1648), 97.3% (1603/1648), and 95.1% (1568/1648), respectively. After analyzing these results, we found that the <50 nm group contained significantly fewer types of proteins than the 50–100 nm and 100–300 nm groups. The 50–100 nm group contained the greatest variety of proteins among the three groups. Interestingly, the percentage of common proteins among the three groups was 15.8% (261/1648). Furthermore, the 261 types of common proteins among the three groups contained five groups: cytoplasmic (115), periplasmic (84), outer membrane (36), inner membrane (4), and extracellular (22). We then focused on membrane proteins in our subsequent LC-MS/MS analyses. As shown in Figure 4b and Table S3, of the 229 total membrane proteins identified, the <50 nm, 50–100 nm, and 100–300 nm groups contributed 17.5% (40/229), 93.9% (215/229), and 91.3% (209/229), respectively. Among these membrane proteins, 17.5% (40/229) were common to both the <50 nm and 50–100 nm groups, 85.2% (195/229) were common to the 50–100 nm and 100–300 nm groups, and 17.5% (40/229) were common to the <50 nm and 100–300 nm groups. Thus, the <50 nm group contained the fewest unique membrane protein types. Finally, we analyzed the common membrane proteins contained in the three groups of EcN-derived OMVs, as shown in Table S3. We identified 36 types of outer membrane proteins and 4 types of inner membrane proteins (including GlpT, Rne, FeoB, and BsmA) among the common membrane proteins in the three groups. Importantly, these common membrane proteins included typical proteins that could play an immunomodulatory role in host cells (such as many outer membrane proteins, including OmpA, OmpC, and OmpF). In summary, these results indicated that EcN-derived OMVs of different sizes presented obvious heterogeneity in terms of the proteins they contain.

### 3.4. Uptake of EcN-Derived OMVs of Varying Sizes by RAW264.7 Macrophages

**Figure 5:**
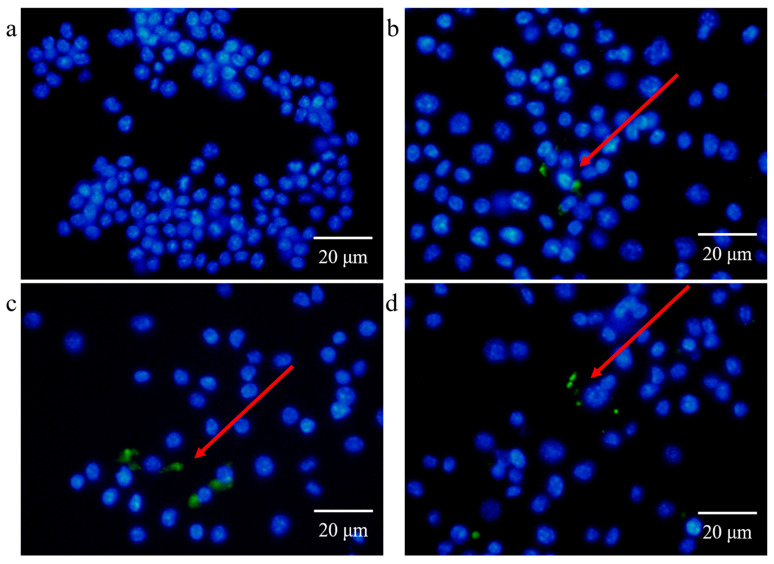
Fluorescence images of the RAW264.7 macrophages incubated with the DiO-labeled EcN derived OMVs of varying sizes after 16 h (scale bars: 20 µm). (**a**) Negative control (without DiO-labeled EcN-derived OMVs) and (**b**) <50 nm, (**c**) 50–100 nm, and (**d**) 100–300 nm EcN-derived OMVs labeled with DiO. The cell membrane dye DiO (green) labels EcN-derived OMVs and Hoechst 33342 (blue) labels nuclei. Representative images from three independent experiments are presented.

In order to investigate the biological activity of EcN-derived OMVs of varying sizes, we performed a fluorescence imaging experiment in which EcN-derived OMVs of varying sizes were taken up by the RAW264.7 macrophages. As shown in Figure 5, EcN-derived OMVs of varying sizes were labeled with cell membrane dye of 3,3′-dioctadecyloxacarbocyanine perchlorate (DiO) and incubated with the RAW264.7 macrophages for 16 h. Phosphate-buffered saline (PBS) was mixed with DiO and purified through ultrafiltration to serve as the negative control group. EcN-derived OMVs of varying sizes were evaluated using fluorescence imaging to determine their ability to be taken up by the recipient RAW264.7 macrophages. As shown in Figure 5a–d, the RAW264.7 cells in the negative control group were free of DiO-labeled EcN-derived OMVs, as no fluorescence was observed in the RAW264.7 cells after 16 h of incubation. In contrast, obvious green fluorescence was observed in the RAW264.7 cells in the experimental groups after 16 h of incubation, indicating the uptake of DiO-labeled EcN-derived OMVs. These results demonstrated that EcN-derived OMVs of varying sizes can be taken up by RAW264.7 macrophages to transmit relevant molecular signals. Moreover, the uptake experiments further indicated that the EcN-derived OMVs of varying sizes showed similar biological activities. These size-independent uptake results highlighted the potential versatility of EcN-derived OMVs as natural nanocarriers for therapeutic applications.

### 3.5. Effect of EcN-Derived OMVs of Varying Sizes on Inflammatory Cytokine Secretion by RAW264.7 Macrophages

**Figure 6:**
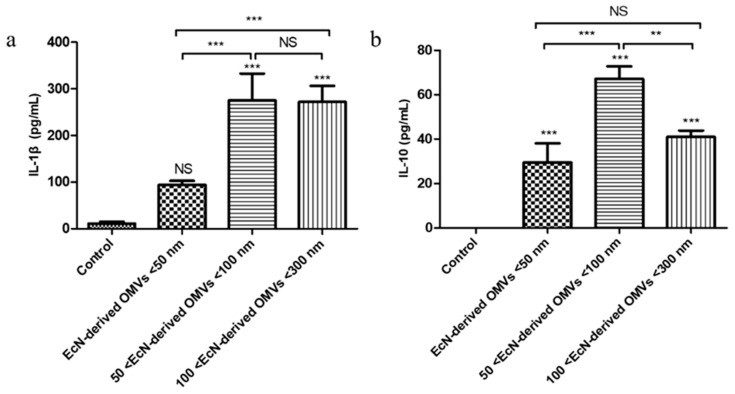
Effect of stimulation with the three sizes of EcN-derived OMVs at 4 µg/mL on the secretion of pro-inflammatory and anti-inflammatory cytokines by RAW264.7 macrophages: (**a**) IL-1β and (**b**) IL-10. The data are presented as the mean ± standard deviations of three independent experiments. ** *p* < 0.01, and *** *p* < 0.001, as determined via one-way analysis of variance (ANOVA).

Research has shown that EcN-derived OMVs can trigger both pro-inflammatory and anti-inflammatory responses in RAW264.7 macrophages [26]. To the best of our knowledge, there have been no reports on the efficacy of EcN-derived OMVs of different sizes on RAW264.7 macrophages. Thus, we co-cultured 4 µg/mL of EcN-derived OMVs of three specific sizes with RAW264.7 macrophages for 16 h and then measured the secreted pro-inflammatory IL-1β and anti-inflammatory IL-10 factors via enzyme-linked immunosorbent assay (ELISA) experiments. The standard ELISA curves are shown in Figure S7, and the results are presented in Figure 6. The experimental results indicated that the secretion level of the pro-inflammatory cytokine IL-1β by RAW264.7 macrophages in the PBS control group was minimal, whereas the anti-inflammatory cytokine IL-10 was undetectable in the cell supernatant. The levels of IL-1β produced by the experimental group of RAW264.7 macrophages were significantly greater (Figure 6a) than those produced by the PBS control group. The IL-1β concentrations in the three EcN-derived OMVs groups (<50 nm, 50–100 nm, and 100–300 nm) were 93.7 ± 8.6 pg/mL, 274.9 ± 58.1 pg/mL, and 271.9 ± 34.1 pg/mL, respectively. Furthermore, the concentration of IL-1β in the <50 nm group was lower than that in the 50–100 nm and 100–300 nm groups. Notably, the concentration of IL-10 secreted by the experimental group of RAW264.7 macrophages increased with EcN-derived OMV size (Figure 6b). The IL-10 concentration was highest in the RAW264.7 macrophages incubated with the 50–100 nm EcN-derived OMVs (67.1 ± 5.7 pg/mL), with the corresponding concentrations in those incubated with the <50 nm and 100–300 nm EcN-derived OMVs being 29.5 ± 8.6 pg/mL and 40.9 ± 2.9 pg/mL, respectively. These results suggest that EcN-derived OMVs of different sizes could induce RAW264.7 macrophages to secrete the pro-inflammatory cytokine IL-1β and the anti-inflammatory cytokine IL-10. Moreover, EcN-derived OMVs of different sizes had different effects on the ability of RAW264.7 macrophages to secrete inflammatory cytokines.

## 4. Discussion

OMVs are extracellular vesicles secreted by Gram-negative bacteria with diameters ranging from 20 to 250 nm. OMVs contain biologically active molecules such as proteins, lipids, and nucleic acids, which are secreted by parent bacteria [35]. Since OMVs can transport substances derived from their parent bacteria, they may serve as effective delivery vehicles for transferring bacterial components to host cells during interactions. This mechanism has the potential to induce an immune response in the host [36]. As a novel secretion system, OMVs have many advantages. First, OMVs can selectively encapsulate the contents of parent bacteria, such as lipids, proteins, and LPS. Second, OMVs can protect their contents from being degraded by extracellular enzymes, ensuring that they can safely reach target cells. Finally, OMVs are equipped with surface ligands that interact with host cell receptors, thereby directly eliciting host immune responses. OMVs have significant potential for application in vaccine research and development, drug delivery systems, and antitumor therapeutics [2,6]. Regarding their use in vaccines, OMVs are highly immunogenic but cannot replicate; thus, they can be used as vaccine antigen candidates as well as vaccine adjuvants [37,38]. Probiotic-derived OMVs can be used as drug carriers to prevent and treat corresponding diseases; they are inanimate substances that benefit the host’s health and can avoid potential harm to the host caused by live bacteria while retaining the beneficial and immunogenic properties of the parent bacteria. Regarding antitumor preparations, when combined with other treatment modalities, OMVs can enhance tumor immunotherapy and prevent tumor recurrence and metastasis [39]. EcN is a Gram-negative probiotic that is widely used in clinical treatment because of its ability to significantly alleviate intestinal diseases [40]. OMVs secreted by EcN serve as carriers of signaling molecules and can trigger pro-inflammatory and anti-inflammatory responses in RAW264.7 macrophages, thereby regulating the biological functions of these cells [26,33]. Nevertheless, the heterogeneity of EcN-derived OMVs of varying sizes has received limited scholarly attention.

Presently, frequently employed techniques for separating OMVs include ultracentrifugation, size-exclusion chromatography (SEC), ultrafiltration, and density gradient centrifugation [5,41]. Each of these methods presents distinct advantages and disadvantages. Notably, ultracentrifugation is a widely utilized technique that separates OMVs from cellular debris, bacteria, and other contaminants on the basis of the applied centrifugal force. However, this method is associated with high equipment costs, and the high centrifugal force may damage the structural integrity and functionality of the OMVs. The SEC method achieves the separation of particles on the basis of size through utilizing porous polymer gel packing. While the SEC method offers high separation purity, it is primarily suitable for the purification of samples with small volumes. In contrast, the ultrafiltration technique employs membranes with varying molecular weight cutoffs to selectively separate and collect vesicles. Ultrafiltration has the highest recovery efficiency. Density gradient centrifugation facilitates the distribution of OMVs across specific density layers via ultra-high-speed centrifugation. This method achieves the highest separation purity, but the operation is cumbersome. This study investigated the heterogeneity of EcN-derived OMVs of different sizes. The main objective was to efficiently obtain EcN-derived OMV samples of different sizes that satisfied the experimental requirements. Conventional methods fail to produce EcN-derived OMVs of different sizes. Inspired by the experimental principles of ultrafiltration, we developed a membrane filtration method to obtain EcN-derived OMVs of varying sizes. In this study, we first used the gradient filtration previously developed by our research group specifically for the separation of EcN-derived OMVs, and used SEC to assess the purity of the EcN-derived OMV samples. The experimental findings indicated that the purity of the EcN-derived OMVs obtained via gradient filtration was comparable to that achieved through SEC. Our approach to optimizing the gradient filtration method, through the incorporation of pretreatment steps and the repeated washing of the samples, proved to be highly effective. We then used the membrane filtration method to separate the high-purity isolated EcN-derived OMVs into three major size groups (<50 nm, 50–100 nm, and 100–300 nm), which were confirmed using TEM and DLS. The significant advantages of the membrane filtration method are that it is fast, convenient, and suitable for achieving industrial-scale yields for mass production, and it maintains the biological functional activity of the isolated OMVs.

OMVs contain biologically active molecules, including proteins, lipids, polysaccharides, DNA, and RNA derived from parent bacterial cells. Elucidating the molecular composition of OMVs is crucial for comprehending their function in bacterial growth or interaction with the host. The analysis of OMV components mainly employs proteomic technologies to elucidate their protein composition. Proteomic research on the OMVs of Gram-negative pathogenic bacteria has identified many protein constituents associated with bacterial pathogenesis and immune modulation [42,43]. In this study, our proteomics results revealed that the isolated EcN-derived OMVs contained 646 total protein types. There is a paucity of published research concerning proteomic analyses of EcN-derived OMVs of different sizes. Nice et al. reported that the *Aggregatibacter actinomycetemcomitans* strain JP2 produces two types of vesicles throughout growth, with varying sizes and toxin compositions [44]. Turner et al. reported that the size of *Helicobacter pylori*-derived OMVs determines their protein content, as fewer and less diverse bacterial proteins are contained within smaller OMVs than those contained within larger OMVs [45]. In addition, there are related reports on the heterogeneity of EVs derived from animal cells. Extracellular vesicles (EVs) released by tumor cells have received considerable attention in recent years, as they contain biomarkers such as tumor-specific proteins and nucleic acids (i.e., RNA and DNA) [46,47]. Liu et al. researched the effect of EV size on protein profiles. They isolated EVs of different sizes from cultured media with 22Rv1 prostate cancer cells via ExoTIC devices with two key pore sizes (30 and 50 nm), and reported that EVs of different sizes contained different protein types [34]. In this study, we used LC-MS/MS analysis to perform proteomic analyses of EcN-derived OMVs of three specific sizes: <50 nm, 50–100 nm, and 100–300 nm. Our results revealed that the <50 nm group contained significantly fewer protein types (262) than the 50–100 nm (1603) and 100–300 nm (1568) groups did. We conducted a categorical analysis of 261 proteins commonly present in EcN-derived OMVs of three specific sizes. They were classified into five groups: cytoplasmic (44.1%, 115/261), periplasmic (32.2%, 84/261), outer membrane (13.8%, 36/261), inner membrane (1.5%, 4/261), and extracellular (8.4%, 22/261). Given that the EcN-derived OMVs of three specific sizes were all secreted by the parent bacteria, it was entirely plausible that they shared certain common proteins. The results indicated that the EcN-derived OMVs of three specific sizes possessed a relatively rich variety of common proteins, which suggested that they might perform similar biological functions. As we found in this study, the EcN-derived OMVs of three specific sizes had obvious therapeutic effects on RAW264.7 macrophages and significantly promoted the secretion of the pro-inflammatory factor IL-1β and the anti-inflammatory factor IL-10 by RAW264.7 macrophages. Although our study provides limited proteomic data on EcN-derived OMVs of three distinct sizes, these experimental findings substantiate that EcN-derived OMVs of varying sizes present differences in protein composition. Future research will involve a comprehensive analysis of proteomic data from EcN-derived OMVs of different sizes, with a focus on investigating the types and characteristics of these differential proteins in greater depth.

Most inflammatory responses result from innate immunity, which is mainly caused through the activation of pro-inflammatory signaling cascades by macrophages and leukocytes. The inflammatory cytokines produced by this cascade comprise small soluble proteins produced by these immune cells; these cytokines regulate the host’s immune and inflammatory responses. Inflammatory cytokines involved in inflammatory responses are mainly divided into two categories, namely, pro-inflammatory factors (e.g., TNF-α, IL-6, and IL-1β) and anti-inflammatory factors (e.g., IL-4 and IL-10) [48]. Among them, IL-1β can stimulate immune cells to secrete pro-inflammatory factors such as TNF-α, which plays a pro-inflammatory role. TNF-α is an important regulatory factor in the initiation of immunity and inflammation. IL-10 is an anti-inflammatory factor that can play an anti-inflammatory role. EcN-derived OMVs can stimulate RAW264.7 macrophages to secrete the pro-inflammatory factors TNF-α, IL-6, and IL-1β, and the anti-inflammatory factor IL-10 [26]. In this study, the concentrations of the pro-inflammatory cytokine IL-1β produced by RAW264.7 macrophages in the three types of EcN-derived OMVs of different sizes (<50 nm, 50–100 nm, and 100–300 nm) were 93.7 ± 8.6 pg/mL, 274.9 ± 58.1 pg/mL, and 271.9 ± 34.1 pg/mL, respectively. Moreover, the concentration of anti-inflammatory IL-10 produced by RAW264.7 macrophages in the 50–100 nm group was the highest (67.1 ± 5.7 pg/mL), and the concentrations of IL-10 in the <50 nm and 100–300 nm groups were 29.5 ± 8.6 pg/mL and 40.9 ± 2.9 pg/mL, respectively. Our results revealed that the therapeutic effects of EcN-derived OMVs of different sizes differed; the 50–100 nm group had a stronger therapeutic effect than the other two groups. We analyzed the reasons for the different experimental phenomena observed regarding the macrophage function mentioned above and speculate that these differences may be attributable to the distinct protein compositions of EcN-derived OMVs of varying sizes. Proteomic analyses of the EcN-derived OMVs of three specific sizes revealed that, in addition to the 261 common proteins shared among the three groups, the 50–100 nm group contained 1341 unique proteins that were distinct from those in the <50 nm group. Similarly, the 100–300 nm group contained 1307 unique proteins that were distinct from those in the <50 nm group. These experimental results suggest that the 50–100 nm and 100–300 nm groups might have stronger therapeutic effects on RAW264.7 macrophages than the <50 nm group. Furthermore, a comparative analysis between the 50–100 nm group and the 100–300 nm group revealed that, in addition to sharing 1523 common proteins, each group also contained distinct proteins. The 50–100 nm group contained 80 unique proteins, whereas the 100–300 nm group contained 45 unique proteins. This variation in protein composition within each group might account for the differential therapeutic effects observed between the 50–100 nm group and the 100–300 nm group on RAW264.7 macrophages. In summary, these differences in macrophage function are consistent with the differences in the proteomic information of the EcN-derived OMVs of varying sizes that we measured. In the macrophage function experiment, we only measured changes in the representative pro-inflammatory cytokine IL-1β and the anti-inflammatory cytokine IL-10. Subsequent experiments should be conducted to further analyze the changes in other pro-inflammatory factors, such as TNF-α, IL-6, and other anti-inflammatory factors, such as IL-4.

Although our experimental findings suggest that EcN-derived OMVs of varying sizes are heterogeneous in terms of protein composition and interactions with RAW264.7 macrophages, additional research is necessary to establish more comprehensive conclusions. Our preliminary proteomic results suggest that EcN-derived OMVs of different sizes present distinct protein compositions. The composition of other components in EcN-derived OMVs of varying sizes, such as lipopolysaccharides, phospholipids, DNA, and RNA, is currently unknown. This study aimed to elucidate the differences in the therapeutic effects of EcN-derived OMVs of varying sizes on RAW264.7 macrophages through examining the distinct protein compositions associated with these EcN-derived OMVs. Importantly, this may represent only one contributing factor. The variations in other constituents contained in EcN-derived OMVs of different sizes may also influence their effects on RAW264.7 macrophages. Further investigation into these unidentified components is necessary in future research. In addition, the therapeutic efficacy of EcN-derived OMVs of varying sizes on macrophages in mouse models remains unclear and requires further validation. However, notwithstanding these limitations, our results provide direct experimental evidence that EcN-derived OMVs of different sizes exhibit heterogeneous properties.

## 5. Conclusions

EcN-derived OMVs play a crucial role in modulating intestinal immune responses and possess significant potential for clinical applications. Few studies have addressed the heterogeneity of differently sized EcN-derived OMVs, significantly limiting research on their clinical applications. In this study, we successfully obtained EcN-derived OMVs of three specific sizes (<50 nm, 50–100 nm, and 100–300 nm) via the membrane filtration method. Among them, the <50 nm group (262) contained fewer types of total proteins than the 50–100 nm (1603) and 100–300 nm (1568) groups. Moreover, in terms of the differential secretion of the pro-inflammatory factor IL-1β and the anti-inflammatory factor IL-10 in RAW264.7 macrophages, the 50–100 nm group presented a stronger effect than the other two groups. Collectively, our findings reveal that the size of EcN-derived OMVs plays a key role in regulating macrophage function and their protein cargo composition. These findings underscore a significant concern regarding the clinical application of research on EcN-derived OMVs, as variations in their size may influence the experimental outcomes. We speculate that variations may exist not only in their protein composition but also in other constituent elements, as well as in the pathways and mechanisms through which EcN-derived OMVs of different sizes interact with the host. Therefore, we conclude that EcN-derived OMVs of varying sizes exhibit heterogeneous characteristics. These findings have fundamental and significant implications that should be considered when examining the role of EcN-derived OMVs in clinical applications.

## Data Availability

The MS proteomics data have been deposited in the ProteomeXchange Consortium (http://proteomecentral.proteomexchange.org (accessed on 1 December 2024)) via the iProX partner repository with the dataset identifier PXD058454.

## Supplementary Materials

The following supporting information can be downloaded at: https://www.mdpi.com/article/10.3390/membranes15050141/s1. Supplementary File S1 (docx): Figure S1: Protein analysis of the *Escherichia coli* Nissle 1917 (EcN)-derived OMVs obtained using two different methods [size-exclusion chromatography (SEC) and gradient filtration] was carried out with sodium dodecyl sulfate–polyacrylamide gel electrophoresis (SDS-PAGE). Figure S2: Effect of stimulation with the EcN-derived OMVs purified via SEC (4 µg/mL) on the secretion of pro-inflammatory and anti-inflammatory cytokines by RAW264.7 macrophages. Figure S3: Protein analysis of EcN-derived OMVs was conducted via SDS-PAGE, with three groups of repeated experiments. Figure S4: Venn diagram showing the 646 common proteins between the four groups of repeated experiments. Figure S5: Schematic of the segmentation of OMVs into three types of different sizes (<50 nm, 50–100 nm, and 100–300 nm). Figure S6: The particle size distributions of the differently sized EcN-derived OMVs were measured via dynamic light scattering (DLS): (a) <50 nm EcN-derived OMVs; (b) 50–100 nm EcN-derived OMVs; (c) 100–300 nm EcN-derived OMVs. Figure S7: Standard curves were used to determine the pro-inflammatory and anti-inflammatory cytokine concentrations via ELISA: standard curves for (a) IL-1β and (b) IL-10. Table S1: EcN-derived OMV proteins identified in this study. Supplementary File S2 (xlsx): Table S2: Comparison of the total proteins contained in the EcN-derived OMVs of three specific sizes (<50 nm, 50–100 nm, and 100–300 nm). Supplementary File S3 (xlsx): Table S3: Comparison of the membrane proteins contained in the EcN-derived OMVs of three specific sizes (<50 nm, 50–100 nm, and 100–300 nm).

## Abbreviations

OMVs	Outer membrane vesicles
LC-MS/MS	Liquid chromatography with tandem mass spectrometry
EcN	*Escherichia coli* Nissle 1917
TEM	Transmission electron microscopy
NTA	Nanoparticle tracking analysis
DLS	Dynamic light scattering
SDS-PAGE	Sodium dodecyl sulfate-polyacrylamide gel electrophoresis
BCA	Bicinchoninic acid
DiO	3,3′-dioctadecyloxacarbocyanine perchlorate
IL-1β	Interleukin-1β
IL-10	Interleukin-10
ELISA	Enzyme-linked immunosorbent assay
PBS	Phosphate-buffered saline
EVs	Extracellular vesicles
SEC	Size-exclusion chromatography
