# Supplementary material for: Separation and Characterization of Heterogeneity Among Various Sizes of Outer Membrane Vesicles Derived from the Probiotic Escherichia coli Nissle 1917
Source: Membranes (Basel). 2025 May 5;15(5):141. doi: 10.3390/membranes15050141 (PMC12113494; doi:10.3390/membranes15050141)
Supplement: Supplementary file 1 [file membranes-15-00141-s001.zip › Supplementary file 1 2025.5.4.pdf]

Supplementary file 1

Separation and characterization of heterogeneity among various sizes of outer membrane vesicles derived from the probiotic *Escherichia coli* Nissle 1917

Ning Li*, Hongbo Xin and Keyu Deng *

The national Engineering Research Center for Bioengineering Drugs and the Technologies, Jiangxi Province Key Laboratory of Bioengineering Drugs, Institute of Translational Medicine, Jiangxi Medical College, Nanchang University, Nanchang, 330031, China; lining@ncu.edu.cn (N.L.); xinhb@ncu.edu.cn (H.X.); dky@ncu.edu.cn (K.D.)

*Correspondence: lining@ncu.edu.cn (N.L.); dky@ncu.edu.cn (K.D.)

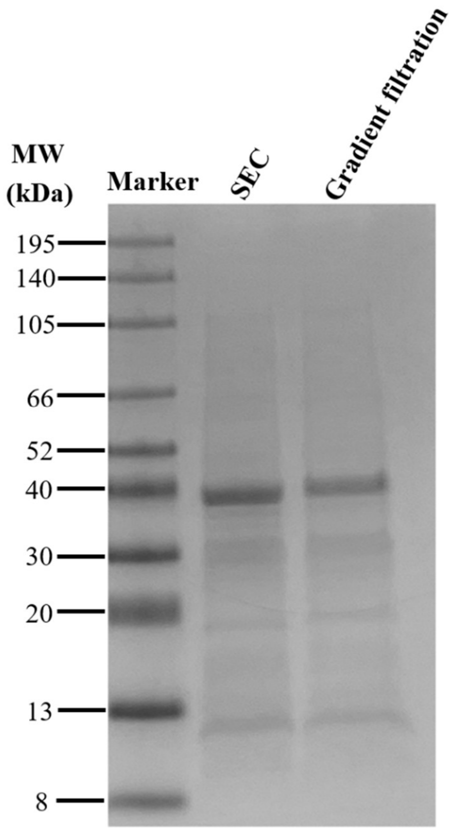

Figure S1. Protein analysis of the *Escherichia coli* Nissle 1917 (EcN)-derived OMVs obtained using the two different methods [size-exclusion chromatography (SEC) and gradient filtration] was carried out with sodium dodecyl sulfate–polyacrylamide gel electrophoresis (SDS–PAGE).

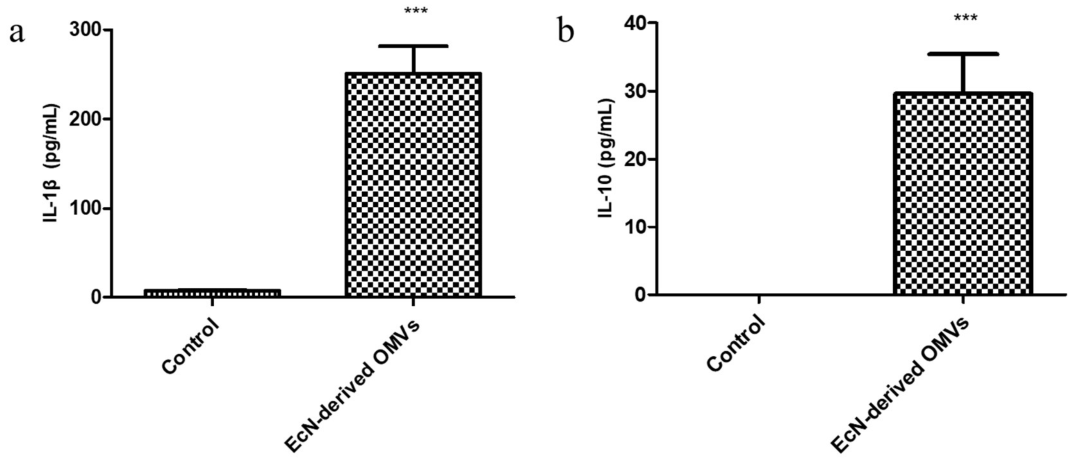

Figure S2. Effect of stimulation with the EcN-derived OMVs purified via SEC (4 μg/mL) on the secretion of pro-inflammatory and anti-inflammatory cytokines by RAW264.7 macrophages; (a) IL-1β; (b) IL-10. Data are represented as means ± SDs of three independent experiments. * $p < 0.05$, and *** $p < 0.001$, as determined using a student's t-test.

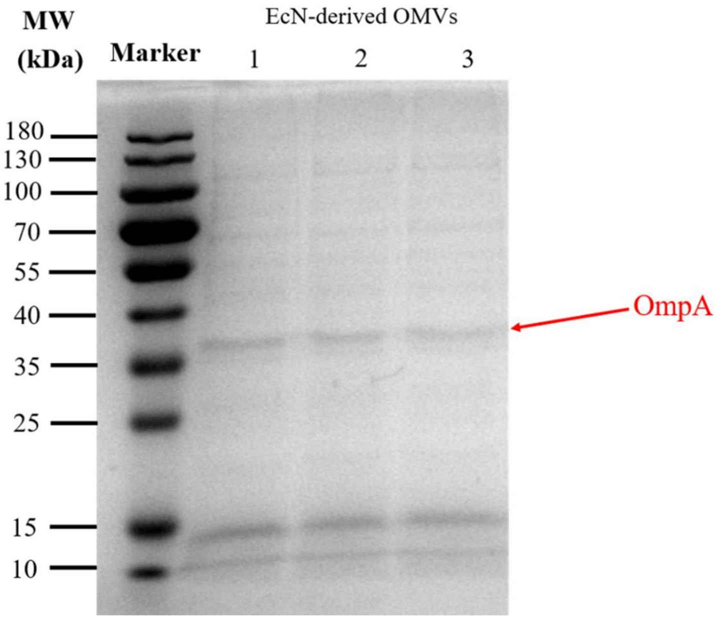

Figure S3. Protein analysis of EcN-derived OMVs was conducted via SDS–PAGE, with three groups of repeated experiments.

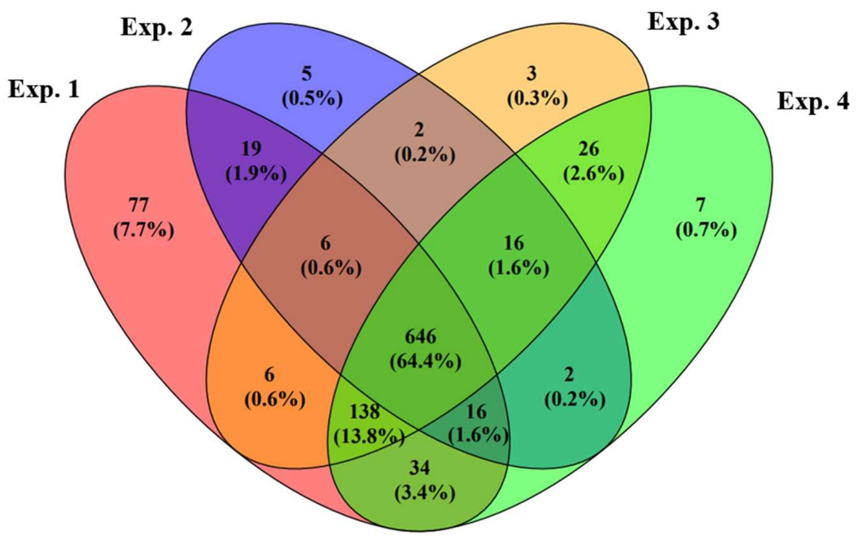

Figure S4. Venn diagram showing the 646 common proteins between the four groups of repeated experiments.

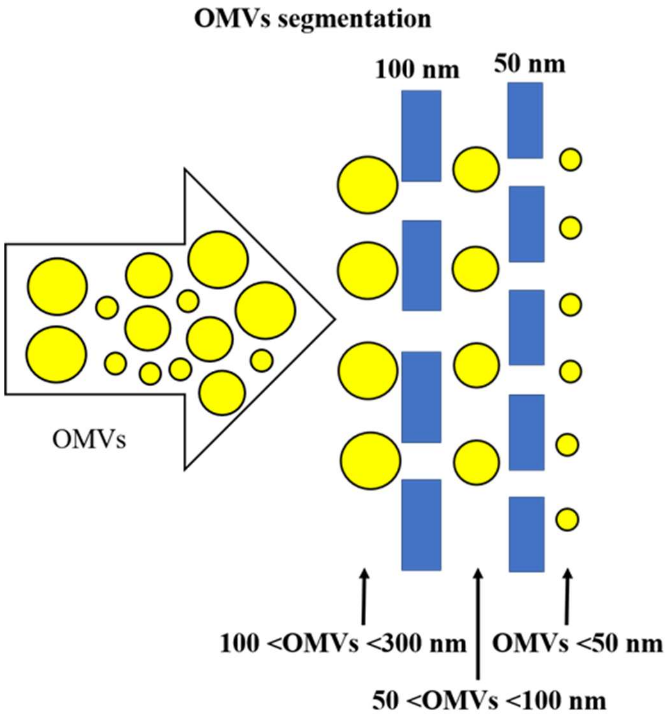

Figure S5. Schematic of the segmentation of OMVs into three types with different sizes (<50 nm, 50–100 nm, and 100–300 nm).

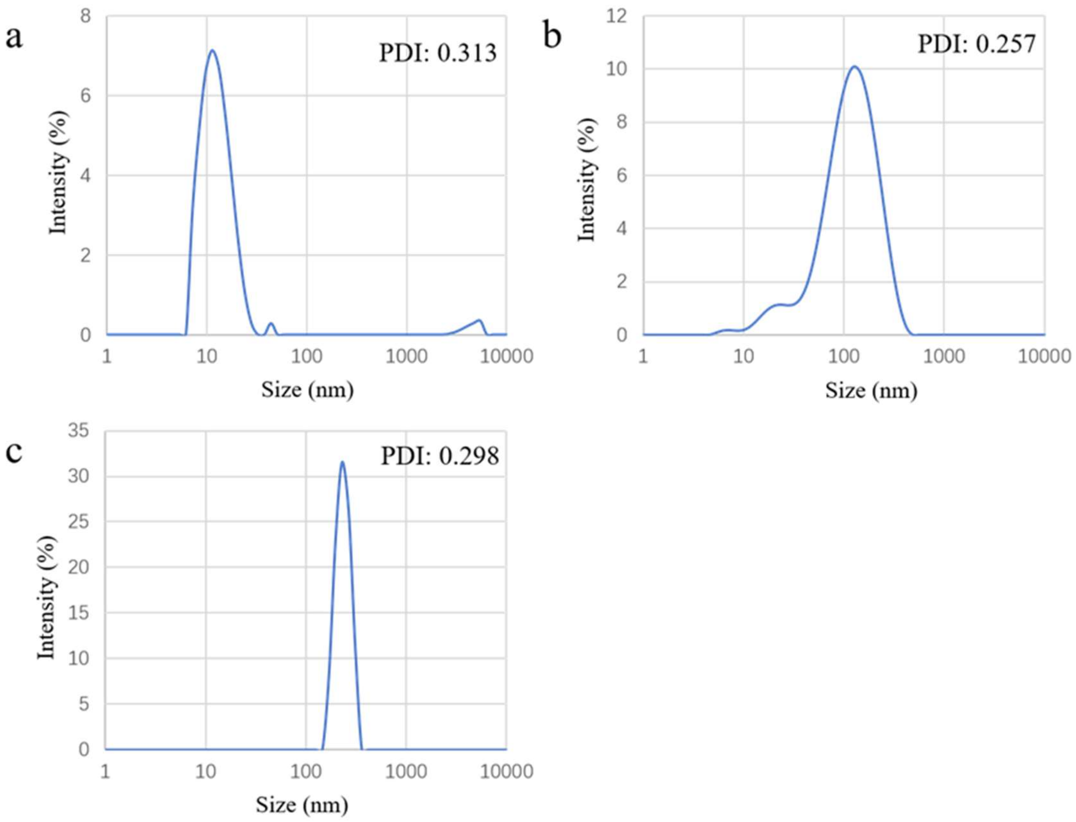

Figure S6. The particle size distributions of the differently sized EcN-derived OMVs were measured via dynamic light scattering (DLS): (a) <50 nm EcN-derived OMVs; (b) 50–100 nm EcN-derived OMVs; (c) 100–300 nm EcN-derived OMVs.

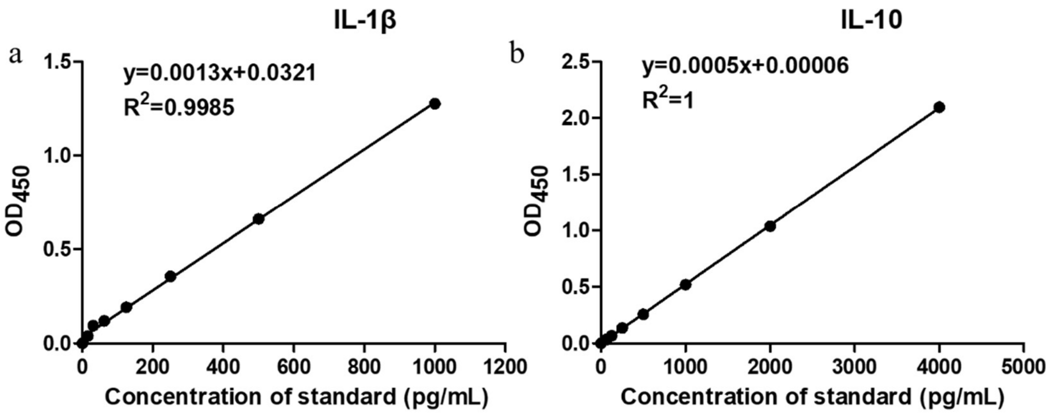

Figure S7. Standard curves were used to determine the pro-inflammatory and anti-inflammatory cytokine concentrations via ELISA: standard curves for (a) IL-1β and (b) IL-10.

Table S1. EcN-derived OMV proteins identified in this study.

**Gene name**	**Protein accession**	**Protein description**	**COG Functional Category**
		**Outer membrane**	

hldD	B1X953	ADP-L-glycero-D-manno-heptose-6-epimerase	Nucleotide transport and metabolism
lamB	B1XC35	Maltoporin	Cell wall/membrane/envelope biogenesis
bamA	B1XD46	Outer membrane protein assembly factor BamA	Cell wall/membrane/envelope biogenesis
hfq	B1XDS3	RNA-binding protein Hfq	Translation, ribosomal structure, and biogenesis
mltC	B1XFC3	Membrane-bound lytic murein transglycosylase C	Cell wall/membrane/envelope biogenesis
metL	P00562	Bifunctional aspartokinase/homoserine dehydrogenase 2	Amino acid transport and metabolism
tolC	P02930	Outer membrane protein TolC	Cell wall/membrane/envelope biogenesis
ompF	P02931	Outer membrane porin F	Cell wall/membrane/envelope biogenesis
fepA	P05825	Ferrienterobactin receptor	Inorganic ion transport and metabolism
btuB	P06129	Vitamin B12 transporter BtuB	Inorganic ion transport and metabolism
fhuA	P06971	Ferrichrome outer membrane transporter/phage receptor	Inorganic ion transport and metabolism
ompC	P06996	Outer membrane porin C	Cell wall/membrane/envelope biogenesis
ompT	P09169	Protease 7	Cell wall/membrane/envelope biogenesis
mipA	P0A908	MltA-interacting protein	Cell wall/membrane/envelope biogenesis
ompA	P0A910	Outer membrane protein A	Cell wall/membrane/envelope biogenesis
pal	P0A912	Peptidoglycan-associated lipoprotein	Cell wall/membrane/envelope biogenesis
ompW	P0A915	Outer membrane protein W	Cell wall/membrane/envelope biogenesis
ompX	P0A917	Outer membrane protein X	Cell wall/membrane/envelope biogenesis
pldA	P0A921	Phospholipase A1	Cell wall/membrane/envelope biogenesis
tsx	P0A927	Nucleoside-specific channel-forming protein Tsx	Cell wall/membrane/envelope biogenesis
fbaB	P0A991	Fructose-bisphosphate aldolase class 1	Nucleotide transport and metabolism
pta	P0A9M8	Phosphate acetyltransferase	Energy production and conversion
zapG	P0ADW3	Z-ring associated protein G	Function unknown
tldD	P0AGG8	Metalloprotease TldD	Function unknown
fadL	P10384	Long-chain fatty acid transport protein	Cell wall/membrane/envelope biogenesis
damX	P11557	Cell division protein DamX	Cell cycle control, cell division, and chromosome partitioning
fecA	P13036	Fe(3+) dicitrate transport protein FecA	Inorganic ion transport and metabolism
cirA	P17315	Colicin I receptor	Inorganic ion transport and metabolism
nmpC	P21420	Putative outer membrane porin protein NmpC	Cell wall/membrane/envelope biogenesis
mliC	P28224	Membrane-bound lysozyme inhibitor of C-type lysozyme	Function unknown
lptD	P31554	LPS-assembly protein LptD	Cell wall/membrane/envelope biogenesis
bcsC	P37650	Cellulose synthase operon protein C	Posttranslational modification, protein turnover, and chaperones
lpoA	P45464	Penicillin-binding protein activator LpoA	Cell wall/membrane/envelope biogenesis
dolP	P64596	Outer membrane lipoprotein DolP	Function unknown
fiu	P75780	Catecholate siderophore receptor Fiu	Inorganic ion transport and metabolism
ybjP	P75818	Uncharacterized lipoprotein YbjP	Cell wall/membrane/envelope biogenesis
pqqU	P76115	Pyrroloquinoline quinone transporter	Inorganic ion transport and metabolism
bamB	P77774	Outer membrane protein assembly factor BamB	Cell wall/membrane/envelope biogenesis
ygeR	Q46798	Uncharacterized lipoprotein YgeR	Cell wall/membrane/envelope biogenesis

Inner membrane

gppA	B1X9Z3	Guanosine-5'-triphosphate,3'-diphosphate pyrophosphatase	Nucleotide transport and metabolism
hflD	B1XA42	High-frequency lysogenization protein HflD	Function unknown
xseA	B1XAY4	Exodeoxyribonuclease 7 large subunit	Replication, recombination, and repair
maeA	B1XE70	NAD-dependent malic enzyme	Energy production and conversion
tdcC	B1XGT1	Threonine/serine transporter TdcC	Amino acid transport and metabolism
mrcB	P02919	Penicillin-binding protein 1B	Cell wall/membrane/envelope biogenesis
proC	P0A9L8	Pyrroline-5-carboxylate reductase	Amino acid transport and metabolism
yagU	P0AAA1	Inner membrane protein YagU	Function unknown
cydA	P0ABJ9	Cytochrome bd-I ubiquinol oxidase subunit 1	Energy production and conversion
hdhA	P0AET8	7alpha-hydroxysteroid dehydrogenase	Lipid transport and metabolism
secG	P0AG99	Protein-export membrane protein SecG	Intracellular trafficking, secretion, and vesicular transport
chrR	P0AGE6	Quinone reductase	Function unknown
ratA	P0AGL5	Ribosome association toxin RatA	Lipid transport and metabolism
rne	P21513	Ribonuclease E	Translation, ribosomal structure, and biogenesis
		**Extracellular**	
ECDH10B_1076	B1X9C5	NAD(P)H dehydrogenase	Function unknown
flgH	B1X9J2	Flagellar L-ring protein	Cell motility
ecpA	B1XE35	Common pilus major fimbrillin subunit EcpA	Function unknown
ygiB	C4ZQV5	UPF0441 protein YgiB	Function unknown
fliC	P04949	Flagellin	Cell motility
slyB	P0A905	Outer membrane lipoprotein SlyB	Cell wall/membrane/envelope biogenesis
mltA	P0A935	Membrane-bound lytic murein transglycosylase A	Cell wall/membrane/envelope biogenesis
ybgS	P0AAV6	Uncharacterized protein YbgS	Function unknown
flgG	P0ABX5	Flagellar basal-body rod protein FlgG	Cell motility
ydcA	P0ACW4	Uncharacterized protein YdcA	Function unknown
nlpD	P0ADA3	Murein hydrolase activator NlpD	Cell cycle control, cell division, and chromosome partitioning
ecnB	P0ADB7	Entericidin B	Function unknown
yjeI	P0AF70	Uncharacterized protein YjeI	Function unknown
ssb	P0AGE0	Single-stranded DNA-binding protein	Replication, recombination, and repair
rlpA	P10100	Endolytic peptidoglycan transglycosylase RlpA	Cell wall/membrane/envelope biogenesis
fliD	P24216	Flagellar hook-associated protein 2	Cell motility
flgL	P29744	Flagellar hook-associated protein 3	Cell motility
flgK	P33235	Flagellar hook-associated protein 1	Cell motility
slp	P37194	Outer membrane protein Slp	Cell wall/membrane/envelope biogenesis
yiaD	P37665	Probable lipoprotein YiaD	Cell wall/membrane/envelope biogenesis
flu	P39180	Antigen 43	Cell wall/membrane/envelope biogenesis
ybhC	P46130	Putative acyl-CoA thioester hydrolase YbhC	Cell wall/membrane/envelope biogenesis
ygdR	P65294	Uncharacterized lipoprotein YgdR	Function unknown
flgE	P75937	Flagellar hook protein FlgE	Cell motility

mlaA	P76506	Intermembrane phospholipid transport system lipoprotein MlaA	Cell wall/membrane/envelope biogenesis
yfeY	P76537	Uncharacterized protein YfeY	Function unknown
gpr	Q46851	L-glyceraldehyde 3-phosphate reductase	Energy production and conversion
		**Periplasmic**	
rplP	B1X6G5	Large ribosomal subunit protein uL16	Translation, ribosomal structure, and biogenesis
rplB	B1X6G8	Large ribosomal subunit protein uL2	Translation, ribosomal structure, and biogenesis
rpsG	B1X6J1	Small ribosomal subunit protein uS7	Translation, ribosomal structure, and biogenesis
tolB	B1X6S1	Tol-Pal system protein TolB	Intracellular trafficking, secretion, and vesicular transport
pckA	B1X750	Phosphoenolpyruvate carboxykinase (ATP)	Nucleotide transport and metabolism
cdd	B1X7N2	Cytidine deaminase	Nucleotide transport and metabolism
lolA	B1X831	Outer-membrane lipoprotein carrier protein	Cell wall/membrane/envelope biogenesis
napA	B1X8A2	Periplasmic nitrate reductase	Energy production and conversion
eco	B1X8A5	Ecotin	Cell wall/membrane/envelope biogenesis
mdoG	B1X9G2	Glucans biosynthesis protein G	Inorganic ion transport and metabolism
flgI	B1X9J3	Flagellar P-ring protein	Cell motility
atpG	B1X9W1	ATP synthase gamma chain	Energy production and conversion
atpF	B1X9W4	ATP synthase subunit b	Energy production and conversion
emtA	B1XAN4	Endo-type membrane-bound lytic murein transglycosylase A	Cell wall/membrane/envelope biogenesis
lolB	B1XAQ0	Outer-membrane lipoprotein LolB	Cell wall/membrane/envelope biogenesis
kdsA	B1XAQ6	2-dehydro-3-deoxyphosphooctonate aldolase	Cell wall/membrane/envelope biogenesis
pepB	B1XAZ8	Peptidase B	Amino acid transport and metabolism
katG	B1XBA8	Catalase-peroxidase	Inorganic ion transport and metabolism
rpsT	B1XBE8	Small ribosomal subunit protein bS20	Translation, ribosomal structure, and biogenesis
rplK	B1XBY5	Large ribosomal subunit protein uL11	Translation, ribosomal structure, and biogenesis
rplA	B1XBY6	Large ribosomal subunit protein uL1	Translation, ribosomal structure, and biogenesis
zapA	B1XEJ6	Cell division protein ZapA	Cell cycle control, cell division, and chromosome partitioning
ynfB	B1XF48	UPF0482 protein YnfB	Function unknown
Mug	B1XG73	G/U mismatch-specific DNA glycosylase	Replication, recombination, and repair
Fis	B1XHN0	DNA-binding protein Fis	Transcription
rpsL	C4ZUJ7	Small ribosomal subunit protein uS12	Translation, ribosomal structure, and biogenesis
lptE	C4ZWC8	LPS-assembly lipoprotein LptE	Cell wall/membrane/envelope biogenesis
leuS	C4ZWC9	Leucine--tRNA ligase	Translation, ribosomal structure, and biogenesis
fabD	D0VE86	Malonyl CoA-acyl carrier protein transacylase	Lipid transport and metabolism
ansB	P00805	L-asparaginase 2	Amino acid transport and metabolism
trpS	P00954	Tryptophan--tRNA ligase	Translation, ribosomal structure, and biogenesis
rbsB	P02925	Ribose import binding protein RbsB	Carbohydrate transport and metabolism
malM	P03841	Maltose operon periplasmic protein	Function unknown
topA	P06612	DNA topoisomerase 1	Replication, recombination, and repair
pfkB	P06999	ATP-dependent 6-phosphofructokinase isozyme 2	Nucleotide transport and metabolism

yfiB	P07021	Putative lipoprotein YfiB	Cell wall/membrane/envelope biogenesis
ushA	P07024	Protein UshA	Nucleotide transport and metabolism
cpdB	P08331	2',3'-cyclic-nucleotide 2'-phosphodiesterase/3'-nucleotidase	Nucleotide transport and metabolism
dacC	P08506	D-alanyl-D-alanine carboxypeptidase DacC	Cell wall/membrane/envelope biogenesis
galE	P09147	UDP-glucose 4-epimerase	Cell wall/membrane/envelope biogenesis
narG	P09152	Respiratory nitrate reductase 1 alpha chain	Energy production and conversion
glpQ	P09394	Glycerophosphodiester phosphodiesterase	Energy production and conversion
blc	P0A901	Outer membrane lipoprotein Blc	Cell wall/membrane/envelope biogenesis
bamC	P0A903	Outer membrane protein assembly factor BamC	Cell wall/membrane/envelope biogenesis
yeaY	P0AA91	Uncharacterized lipoprotein YeaY	Cell wall/membrane/envelope biogenesis
ybiS	P0AAX8	Probable L,D-transpeptidase YbiS	Cell wall/membrane/envelope biogenesis
lpoB	P0AB38	Penicillin-binding protein activator LpoB	Cell wall/membrane/envelope biogenesis
ymgD	P0AB46	Uncharacterized protein YmgD	Function unknown
fbaA	P0AB71	Fructose-bisphosphate aldolase class 2	Nucleotide transport and metabolism
garR	P0ABQ2	2-hydroxy-3-oxopropionate reductase	Lipid transport and metabolism
surA	P0ABZ6	Chaperone SurA	Posttranslational modification, protein turnover, chaperones
bamD	P0AC02	Outer membrane protein assembly factor BamD	Cell wall/membrane/envelope biogenesis
fumA	P0AC33	Fumarate hydratase class I	Energy production and conversion
frdB	P0AC47	Fumarate reductase iron-sulfur subunit	Energy production and conversion
hupA	P0ACF0	DNA-binding protein HU-alpha	Replication, recombination, and repair
yfhG	P0AD44	Uncharacterized protein YfhG	Cell motility
yajG	P0ADA5	Uncharacterized lipoprotein YajG	Cell wall/membrane/envelope biogenesis
osmE	P0ADB1	Osmotically inducible putative lipoprotein OsmE	Cell wall/membrane/envelope biogenesis
yidQ	P0ADM4	Uncharacterized protein YidQ	Function unknown
ygaM	P0ADQ7	Uncharacterized protein YgaM	Function unknown
yggE	P0ADS6	Uncharacterized protein YggE	Function unknown
lptA	P0ADV1	Lipopolysaccharide export system protein LptA	Function unknown
mlaC	P0ADV7	Intermembrane phospholipid transport system binding protein MlaC	Secondary metabolites biosynthesis, transport and catabolism
dacA	P0AEB2	D-alanyl-D-alanine carboxypeptidase DacA	Cell wall/membrane/envelope biogenesis
dcrB	P0AEE1	Inner membrane lipoprotein DcrB	Function unknown
mglB	P0AEE5	D-galactose/methyl-galactoside binding periplasmic protein MglB	Carbohydrate transport and metabolism
dsbA	P0AEG4	Disulfide interchange protein DsbA	Posttranslational modification, protein turnover, chaperones
dsbC	P0AEG6	Disulfide interchange protein DsbC	Posttranslational modification, protein turnover, chaperones
tcyJ	P0AEM9	L-cystine-binding protein TcyJ	Amino acid transport and metabolism
glcG	P0AEQ1	Protein GlcG	Function unknown
glnH	P0AEQ3	Glutamine-binding periplasmic protein	Amino acid transport and metabolism
hdeB	P0AET2	Acid stress chaperone HdeB	Cell wall/membrane/envelope biogenesis
hisJ	P0AEU0	Histidine-binding periplasmic protein	Amino acid transport and metabolism

skp	P0AEU7	Chaperone protein Skp	Cell wall/membrane/envelope biogenesis
malE	P0AEX9	Maltose/maltodextrin-binding periplasmic protein	Inorganic ion transport and metabolism
osmY	P0AFH8	Osmotically inducible protein Y	Function unknown
potD	P0AFK9	Spermidine/putrescine-binding periplasmic protein	Inorganic ion transport and metabolism
psiF	P0AFM4	Phosphate starvation-inducible protein PsiF	Function unknown
pstS	P0AG82	Phosphate-binding protein PstS	Inorganic ion transport and metabolism
ffh	P0AGD7	Signal recognition particle protein	Intracellular trafficking, secretion, and vesicular transport
degP	P0C0V0	Periplasmic serine endoprotease DegP	Posttranslational modification, protein turnover, and chaperones
fumB	P14407	Fumarate hydratase class I	Energy production and conversion
agp	P19926	Glucose-1-phosphatase	Function unknown
speA	P21170	Biosynthetic arginine decarboxylase	Coenzyme transport and metabolism
katE	P21179	Catalase HPII	Inorganic ion transport and metabolism
oppA	P23843	Periplasmic oligopeptide-binding protein OppA	Amino acid transport and metabolism
dppA	P23847	Dipeptide-binding protein	Amino acid transport and metabolism
acs	P27550	Acetyl-coenzyme A synthetase	Nucleotide transport and metabolism
metQ	P28635	D-methionine-binding lipoprotein MetQ	Inorganic ion transport and metabolism
ftsN	P29131	Cell division protein FtsN	Cell cycle control, cell division, and chromosome partitioning
yedD	P31063	Uncharacterized lipoprotein YedD	Cell wall/membrane/envelope biogenesis
bglX	P33363	Periplasmic beta-glucosidase	Carbohydrate transport and metabolism
nikA	P33590	Nickel-binding periplasmic protein	Amino acid transport and metabolism
pgm	P36938	Phosphoglucomutase	Carbohydrate transport and metabolism
modA	P37329	Molybdate-binding protein ModA	Inorganic ion transport and metabolism
yhjJ	P37648	Protein YhjJ	Function unknown
gltI	P37902	Glutamate/aspartate import solute-binding protein	Amino acid transport and metabolism
nfsB	P38489	Oxygen-insensitive NAD(P)H nitroreductase	Energy production and conversion
degQ	P39099	Periplasmic pH-dependent serine endoprotease DegQ	Cell wall/membrane/envelope biogenesis
nlpE	P40710	Lipoprotein NlpE	Cell wall/membrane/envelope biogenesis
mltB	P41052	Membrane-bound lytic murein transglycosylase B	Cell wall/membrane/envelope biogenesis
fkpA	P45523	FKBP-type peptidyl-prolyl cis-trans isomerase FkpA	Posttranslational modification, protein turnover, and chaperones
cpoB	P45955	Cell division coordinator CpoB	Cell cycle control, cell division, and chromosome partitioning
hslJ	P52644	Heat shock protein HslJ	Posttranslational modification, protein turnover, and chaperones
ydbK	P52647	Probable pyruvate-flavodoxin oxidoreductase	Energy production and conversion
amiC	P63883	N-acetylmuramoyl-L-alanine amidase AmiC	Cell wall/membrane/envelope biogenesis
ydcL	P64451	Uncharacterized lipoprotein YdcL	Cell wall/membrane/envelope biogenesis
yoaF	P64493	Uncharacterized protein YoaF	Function unknown
yhcN	P64614	Uncharacterized protein YhcN	Function unknown
rcsF	P69411	Outer membrane lipoprotein RcsF	Cell wall/membrane/envelope biogenesis
lpp	P69776	Major outer membrane lipoprotein Lpp	Cell wall/membrane/envelope biogenesis

yahO	P75694	Uncharacterized protein YahO	Function unknown
ydgH	P76177	Protein YdgH	Function unknown
yoaA	P76257	ATP-dependent DNA helicase YoaA	Transcription
flhE	P76297	Flagellar protein FlhE	Cell motility
ydeN	P77318	Uncharacterized sulfatase YdeN	Inorganic ion transport and metabolism
borD	P77330	Prophage lipoprotein Bor homolog	Function unknown
ybaY	P77717	Uncharacterized lipoprotein YbaY	Function unknown
spy	P77754	Periplasmic chaperone Spy	Cell motility
		**Cytoplasmic**	
tufA	A0A0M3KKV1	Elongation factor Tu	Translation, ribosomal structure, and biogenesis
arcA2	A5PFK0	Disrupted ArcA	Transcription
dcyD	B1X677	D-cysteine desulfhydrase	Amino acid transport and metabolism
rsmB	B1X6E1	Ribosomal RNA small subunit methyltransferase B	Translation, ribosomal structure, and biogenesis
rplQ	B1X6E6	Large ribosomal subunit protein bL17	Translation, ribosomal structure, and biogenesis
rpsD	B1X6E8	Small ribosomal subunit protein uS4	Translation, ribosomal structure and biogenesis
rpsK	B1X6E9	Small ribosomal subunit protein uS11	Translation, ribosomal structure, and biogenesis
rpmJ1	B1X6F1	Large ribosomal subunit protein bL36A	Translation, ribosomal structure, and biogenesis
rplO	B1X6F3	Large ribosomal subunit protein uL15	Translation, ribosomal structure, and biogenesis
rplR	B1X6F6	Large ribosomal subunit protein uL18	Translation, ribosomal structure, and biogenesis
rplF	B1X6F7	Large ribosomal subunit protein uL6	Translation, ribosomal structure, and biogenesis
rpsH	B1X6F8	Small ribosomal subunit protein uS8	Translation, ribosomal structure, and biogenesis
rpsN	B1X6F9	Small ribosomal subunit protein uS14	Translation, ribosomal structure, and biogenesis
rplE	B1X6G0	Large ribosomal subunit protein uL5	Translation, ribosomal structure, and biogenesis
rplN	B1X6G2	Large ribosomal subunit protein uL14	Translation, ribosomal structure, and biogenesis
rplV	B1X6G6	Large ribosomal subunit protein uL22	Translation, ribosomal structure, and biogenesis
rplD	B1X6H0	Large ribosomal subunit protein uL4	Translation, ribosomal structure, and biogenesis
rplC	B1X6H1	Large ribosomal subunit protein uL3	Translation, ribosomal structure, and biogenesis
rpsJ	B1X6H2	Small ribosomal subunit protein uS10	Translation, ribosomal structure, and biogenesis
fusA	B1X6J0	Elongation factor G	Translation, ribosomal structure, and biogenesis
nagB	B1X6L1	Glucosamine-6-phosphate deaminase	Nucleotide transport and metabolism
glnS	B1X6L3	Glutamine--tRNA ligase	Translation, ribosomal structure, and biogenesis
sucC	B1X6Q8	Succinate--CoA ligase	Nucleotide transport and metabolism
hisG	B1X6V6	ATP phosphoribosyltransferase	Nucleotide transport and metabolism
cysG	B1X716	Siroheme synthase	Coenzyme transport and metabolism
aroB	B1X736	3-dehydroquinate synthase	Amino acid transport and metabolism
hslO	B1X748	33 kDa chaperonin	Posttranslational modification, protein turnover, and chaperones
glgA	B1X774	Glycogen synthase	Nucleotide transport and metabolism
glgC	B1X775	Glucose-1-phosphate adenylyltransferase	Nucleotide transport and metabolism
gpmA	B1X786	2,3-bisphosphoglycerate-dependent phosphoglycerate mutase	Nucleotide transport and metabolism
dps	B1X7E2	DNA protection during starvation protein	Inorganic ion transport and metabolism
folE	B1X7P1	GTP cyclohydrolase 1	Nucleotide transport and metabolism

serS	B1X833	Serine--tRNA ligase	Translation, ribosomal structure, and biogenesis
serC	B1X847	Phosphoserine aminotransferase	Coenzyme transport and metabolism
glpB	B1X8D6	Anaerobic glycerol-3-phosphate dehydrogenase subunit B	Energy production and conversion
glyS	B1X8H5	Glycine--tRNA ligase beta subunit	Translation, ribosomal structure, and biogenesis
mukF	B1X8M6	Chromosome partition protein MukF	Cell cycle control, cell division, and chromosome partitioning
mukB	B1X8M8	Chromosome partition protein MukB	Cell cycle control, cell division, and chromosome partitioning
rlmL	B1X8Q2	Ribosomal RNA large subunit methyltransferase K/L	Translation, ribosomal structure, and biogenesis
cbpA	B1X8V5	Curved DNA-binding protein	Posttranslational modification, protein turnover, and chaperones
ECDH10B_2409	B1X8W2	CinA-like protein	Function unknown
arnB	B1X8W6	UDP-4-amino-4-deoxy-L-arabinose--oxoglutarate aminotransferase	Cell wall/membrane/envelope biogenesis
nuoB	B1X8Z9	NADH-quinone oxidoreductase subunit B	Energy production and conversion
accD	B1X927	Acetyl-coenzyme A carboxylase carboxyl transferase subunit beta	Lipid transport and metabolism
pdxB	B1X931	Erythronate-4-phosphate dehydrogenase	Coenzyme transport and metabolism
secB	B1X943	Protein-export protein SecB	Intracellular trafficking, secretion, and vesicular transport
tdh	B1X950	L-threonine 3-dehydrogenase	Amino acid transport and metabolism
dut	B1X974	Deoxyuridine 5'-triphosphate nucleotidohydrolase	Nucleotide transport and metabolism
rpoZ	B1X982	DNA-directed RNA polymerase subunit omega	Transcription
fadJ	B1X9L4	Fatty acid oxidation complex subunit alpha	Lipid transport and metabolism
fadI	B1X9L5	3-ketoacyl-CoA thiolase	Lipid transport and metabolism
glk	B1X9R0	Glucokinase	Nucleotide transport and metabolism
tnaA	B1X9T7	Tryptophanase	Amino acid transport and metabolism
glmU	B1X9V8	Bifunctional protein GlmU	Cell wall/membrane/envelope biogenesis
atpD	B1X9W0	ATP synthase subunit beta	Nucleotide transport and metabolism
atpA	B1X9W2	ATP synthase subunit alpha	Nucleotide transport and metabolism
mnmG	B1X9W9	tRNA uridine 5-carboxymethylaminomethyl modification enzyme MnmG	Cell cycle control, cell division, and chromosome partitioning
asnA	B1X9X2	Aspartate--ammonia ligase	Nucleotide transport and metabolism
rbsD	B1X9X5	D-ribose pyranase	Carbohydrate transport and metabolism
ilvC	B1X9Z0	Ketol-acid reductoisomerase	Amino acid transport and metabolism
rhlB	B1X9Z4	ATP-dependent RNA helicase RhlB	Translation, ribosomal structure, and biogenesis
acpP	B1XA04	Acyl carrier protein	Lipid transport and metabolism
pepT	B1XA37	Peptidase T	Amino acid transport and metabolism
dadA	B1XA76	D-amino acid dehydrogenase	Amino acid transport and metabolism
purC	B1XAE8	Phosphoribosylaminoimidazole-succinocarboxamide synthase	Nucleotide transport and metabolism

ubiE	B1XAJ7	Ubiquinone/menaquinone biosynthesis C-methyltransferase UbiE	Coenzyme transport and metabolism
pepQ	B1XAK9	Xaa-Pro dipeptidase	Amino acid transport and metabolism
upp	B1XAX3	Uracil phosphoribosyltransferase	Nucleotide transport and metabolism
guaA	B1XAY2	GMP synthase	Nucleotide transport and metabolism
hisS	B1XAY9	Histidine--tRNA ligase	Translation, ribosomal structure, and biogenesis
ispG	B1XAZ0	4-hydroxy-3-methylbut-2-en-1-yl diphosphate synthase	Lipid transport and metabolism
rlmN	B1XAZ2	Dual-specificity RNA methyltransferase RlmN	Translation, ribosomal structure, and biogenesis
hscA	B1XB01	Chaperone protein HscA	Posttranslational modification, protein turnover, and chaperones
iscS	B1XB05	Cysteine desulfurase IscS	Amino acid transport and metabolism
glyA	B1XB26	Serine hydroxymethyltransferase	Amino acid transport and metabolism
rnc	B1XB41	Ribonuclease 3	Translation, ribosomal structure, and biogenesis
lepA	B1XB43	Elongation factor 4	Translation, ribosomal structure, and biogenesis
pfkA	B1XB82	ATP-dependent 6-phosphofructokinase isozyme 1	Nucleotide transport and metabolism
tpiA	B1XB85	Triosephosphate isomerase	Nucleotide transport and metabolism
glpK	B1XB92	Glycerol kinase	Nucleotide transport and metabolism
hslU	B1XB97	ATP-dependent protease ATPase subunit HslU	Posttranslational modification, protein turnover, and chaperones
ppc	B1XBC1	Phosphoenolpyruvate carboxylase	Coenzyme transport and metabolism
argH	B1XBC5	Argininosuccinate lyase	Amino acid transport and metabolism
yaaA	B1XBD1	UPF0246 protein YaaA	Function unknown
dnaJ	B1XBE0	Chaperone protein DnaJ	Posttranslational modification, protein turnover, and chaperones
ileS	B1XBF1	Isoleucine--tRNA ligase	Translation, ribosomal structure, and biogenesis
dapB	B1XBF6	4-hydroxy-tetrahydrodipicolinate reductase	Amino acid transport and metabolism
trpB	B1XBL0	Tryptophan synthase beta chain	Amino acid transport and metabolism
pyrF	B1XBN2	Orotidine 5'-phosphate decarboxylase	Nucleotide transport and metabolism
rnb	B1XBN7	Exoribonuclease 2	Translation, ribosomal structure, and biogenesis
grcA	B1XBQ6	Autonomous glycyl radical cofactor	Function unknown
rplS	B1XBS8	Large ribosomal subunit protein bL19	Translation, ribosomal structure, and biogenesis
rpsP	B1XBT1	Small ribosomal subunit protein bS16	Translation, ribosomal structure, and biogenesis
smpB	B1XBU0	SsrA-binding protein	Translation, ribosomal structure, and biogenesis
glaH	B1XBW5	Glutarate 2-hydroxylase	Energy production and conversion
sthA	B1XBX3	Soluble pyridine nucleotide transhydrogenase	Energy production and conversion
rplJ	B1XBY7	Large ribosomal subunit protein uL10	Translation, ribosomal structure, and biogenesis
rplL	B1XBY8	Large ribosomal subunit protein bL12	Translation, ribosomal structure, and biogenesis
rpoB	B1XBY9	DNA-directed RNA polymerase subunit beta	Transcription
rpoC	B1XBZ0	DNA-directed RNA polymerase subunit beta'	Transcription
hemE	B1XC00	Uroporphyrinogen decarboxylase	Coenzyme transport and metabolism
purH	B1XC09	Bifunctional purine biosynthesis protein PurH	Nucleotide transport and metabolism
pgi	B1XC24	Glucose-6-phosphate isomerase	Nucleotide transport and metabolism
rsmH	B1XC59	Ribosomal RNA small subunit methyltransferase H	Translation, ribosomal structure, and biogenesis
murC	B1XC68	UDP-N-acetylmuramate--L-alanine ligase	Cell wall/membrane/envelope biogenesis

secA	B1XC75	Protein translocase subunit SecA	Intracellular trafficking, secretion, and vesicular transport
guaC	B1XC80	GMP reductase	Nucleotide transport and metabolism
alaS	B1XCM5	Alanine--tRNA ligase	Translation, ribosomal structure, and biogenesis
mutS	B1XCR0	DNA mismatch repair protein MutS	Replication, recombination, and repair
hemL	B1XCR0	DNA mismatch repair protein MutS	Coenzyme transport and metabolism
mtnN	B1XD29	5'-methylthioadenosine/S-adenosylhomocysteine nucleosidase	Nucleotide transport and metabolism
dapD	B1XD35	2,3,4,5-tetrahydropyridine-2,6-dicarboxylate N-succinyltransferase	Amino acid transport and metabolism
rpsB	B1XD38	Small ribosomal subunit protein uS2	Translation, ribosomal structure, and biogenesis
fabZ	B1XD49	3-hydroxyacyl	Lipid transport and metabolism
lpxA	B1XD50	Acyl-UDP-N-acetylglucosamine O-acyltransferase	Cell wall/membrane/envelope biogenesis
accA	B1XD54	Acetyl-coenzyme A carboxylase carboxyl transferase subunit alpha	Lipid transport and metabolism
proS	B1XD64	Proline--tRNA ligase	Translation, ribosomal structure, and biogenesis
patD	B1XDF5	Gamma-aminobutyraldehyde dehydrogenase	Energy production and conversion
eno	B1XDI9	Enolase	Nucleotide transport and metabolism
pyrG	B1XDJ0	CTP synthase	Nucleotide transport and metabolism
fucI	B1XDL1	L-fucose isomerase	Carbohydrate transport and metabolism
fucU	B1XDL3	L-fucose mutarotase	Carbohydrate transport and metabolism
groEL	B1XDP7	Chaperonin GroEL	Posttranslational modification, protein turnover, and chaperones
purA	B1XDS8	Adenylosuccinate synthetase	Nucleotide transport and metabolism
rpsF	B1XDV1	Small ribosomal subunit protein bS6	Translation, ribosomal structure, and biogenesis
rpsR	B1XDV2	Small ribosomal subunit protein bS18	Translation, ribosomal structure, and biogenesis
rplI	B1XDV3	Large ribosomal subunit protein bL9	Translation, ribosomal structure, and biogenesis
proB	B1XDY5	Glutamate 5-kinase	Nucleotide transport and metabolism
proA	B1XDY6	Gamma-glutamyl phosphate reductase	Amino acid transport and metabolism
ygfZ	B1XDY6	Gamma-glutamyl phosphate reductase	Function unknown
gcvP	B1XEJ0	Glycine dehydrogenase	Amino acid transport and metabolism
epd	B1XEL0	D-erythrose-4-phosphate dehydrogenase	Coenzyme transport and metabolism
fbp	B1XEL4	Fructose-1,6-bisphosphatase class 1	Carbohydrate transport and metabolism
pyrI	B1XEM5	Aspartate carbamoyltransferase regulatory chain	Nucleotide transport and metabolism
pyrB	B1XEM6	Aspartate carbamoyltransferase catalytic subunit	Nucleotide transport and metabolism
pepA	B1XEN9	Probable cytosol aminopeptidase	Amino acid transport and metabolism
betB	B1XET7	Betaine aldehyde dehydrogenase	Energy production and conversion
rdgC	B1XEY2	Recombination-associated protein RdgC	Translation, ribosomal structure, and biogenesis
tgt	B1XEZ4	Queuine tRNA-ribosyltransferase	Nucleotide transport and metabolism
dxs	B1XF08	1-deoxy-D-xylulose-5-phosphate synthase	Nucleotide transport and metabolism
thiI	B1XF11	tRNA sulfurtransferase	Nucleotide transport and metabolism
metK	B1XFA4	S-adenosylmethionine synthase	Coenzyme transport and metabolism
prfC	B1XFI4	Peptide chain release factor 3	Translation, ribosomal structure, and biogenesis
deoA	B1XFJ2	Thymidine phosphorylase	Nucleotide transport and metabolism

deoD	B1XFJ4	Purine nucleoside phosphorylase DeoD-type	Nucleotide transport and metabolism
gpmB	B1XFK5	Probable phosphoglycerate mutase GpmB	Nucleotide transport and metabolism
tig	B1XFM4	Trigger factor	Cell cycle control, cell division, and chromosome partitioning
clpX	B1XFM6	ATP-dependent Clp protease ATP-binding subunit ClpX	Posttranslational modification, protein turnover, and chaperones
hemH	B1XFR2	Ferrochelatase	Coenzyme transport and metabolism
tyrS	B1XFU9	Tyrosine--tRNA ligase	Translation, ribosomal structure, and biogenesis
ihfA	B1XG19	Integration host factor subunit alpha	Transcription
pheS	B1XG21	Phenylalanine--tRNA ligase alpha subunit	Translation, ribosomal structure, and biogenesis
rplT	B1XG23	Large ribosomal subunit protein bL20	Translation, ribosomal structure, and biogenesis
ribB	B1XG46	3,4-dihydroxy-2-butanone 4-phosphate synthase	Coenzyme transport and metabolism
hldE	B1XG57	Bifunctional protein HldE	Nucleotide transport and metabolism
rpsU	B1XG70	Small ribosomal subunit protein bS21	Translation, ribosomal structure, and biogenesis
rlmG	B1XG88	Ribosomal RNA large subunit methyltransferase G	Translation, ribosomal structure, and biogenesis
uxaC	B1XG96	Uronate isomerase	Carbohydrate transport and metabolism
thrS	B1XGI1	Threonine--tRNA ligase	Translation, ribosomal structure, and biogenesis
garL	B1XGT9	5-keto-4-deoxy-D-glucarate aldolase	Carbohydrate transport and metabolism
pnp	B1XGX6	Polyribonucleotide nucleotidyltransferase	Translation, ribosomal structure, and biogenesis
rpsO	B1XGX7	Small ribosomal subunit protein uS15	Translation, ribosomal structure, and biogenesis
infB	B1XGY0	Translation initiation factor IF-2	Translation, ribosomal structure, and biogenesis
argG	B1XGY3	Argininosuccinate synthase	Nucleotide transport and metabolism
glmM	B1XGY6	Phosphoglucosamine mutase	Carbohydrate transport and metabolism
rlmE	B1XGY9	Ribosomal RNA large subunit methyltransferase E	Translation, ribosomal structure, and biogenesis
proQ	B1XH99	RNA chaperone ProQ	Signal transduction mechanisms
aspS	B1XHD4	Aspartate--tRNA ligase	Translation, ribosomal structure, and biogenesis
obg	B1XHF9	GTPase Obg	Function unknown
nanA	B1XHJ8	N-acetylneuraminate lyase	Coenzyme transport and metabolism
rpsI	B1XHK3	Small ribosomal subunit protein uS9	Translation, ribosomal structure, and biogenesis
rplM	B1XHK4	Large ribosomal subunit protein uL13	Translation, ribosomal structure, and biogenesis
mdh	B1XHK9	Malate dehydrogenase	Energy production and conversion
argR	B1XHL0	Arginine repressor	Transcription
lacZ	B8LFD6	Beta-galactosidase	Carbohydrate transport and metabolism
rapA	C4ZPY3	RNA polymerase-associated protein RapA	Transcription
murA	C4ZSS8	UDP-N-acetylglucosamine 1-carboxyvinyltransferase	Cell wall/membrane/envelope biogenesis
uxuA	C4ZT10	Mannonate dehydratase	Carbohydrate transport and metabolism
rpsC	C4ZUG9	Small ribosomal subunit protein uS3	Translation, ribosomal structure, and biogenesis
lipA	C4ZWB5	Lipoyl synthase	Coenzyme transport and metabolism
rihA	C4ZWD8	Pyrimidine-specific ribonucleoside hydrolase RihA	Nucleotide transport and metabolism
glyQ	C4ZXF0	Glycine--tRNA ligase alpha subunit	Translation, ribosomal structure, and biogenesis
infC	C4ZYI0	Translation initiation factor IF-3	Translation, ribosomal structure, and biogenesis
recA	C4ZYU4	Protein RecA	Replication, recombination, and repair
ilvD	C4ZZ41	Dihydroxy-acid dehydratase	Amino acid transport and metabolism
gcvT	C5A0H7	Aminomethyltransferase	Amino acid transport and metabolism

fabB	D0VEX1	3-oxoacyl-[acyl-carrier-protein] synthase 1	Lipid transport and metabolism
fabF	D0VEX2	3-oxoacyl-[acyl-carrier-protein] synthase 2	Lipid transport and metabolism
fabG	D0VEX3	3-oxoacyl-[acyl-carrier-protein] reductase	Lipid transport and metabolism
fabH	D0VEX4	Beta-ketoacyl-[acyl-carrier-protein] synthase III	Lipid transport and metabolism
fabI	D0VEX5	Enoyl-[acyl-carrier-protein] reductase	Lipid transport and metabolism
gutQ	D0VEX7	Arabinose 5-phosphate isomerase	Nucleotide transport and metabolism
pssA	D0VEY9	Phosphatidylserine synthase	Lipid transport and metabolism
accC	D0VEZ1	Biotin carboxylase	Lipid transport and metabolism
gnd	P00350	6-phosphogluconate dehydrogenase	Nucleotide transport and metabolism
frdA	P00363	Fumarate reductase flavoprotein subunit	Energy production and conversion
gdhA	P00370	NADP-specific glutamate dehydrogenase	Amino acid transport and metabolism
ndh	P00393	Type II NADH quinone oxidoreductase	Energy production and conversion
nrdA	P00452	Ribonucleoside-diphosphate reductase 1 subunit alpha	Nucleotide transport and metabolism
malP	P00490	Maltodextrin phosphorylase	Carbohydrate transport and metabolism
aspC	P00509	Aspartate aminotransferase	Amino acid transport and metabolism
thrA	P00561	Bifunctional aspartokinase/homoserine dehydrogenase 1	Amino acid transport and metabolism
rpoD	P00579	RNA polymerase sigma factor RpoD	Transcription
polA	P00582	DNA polymerase I	Replication, recombination, and repair
thrC	P00934	Threonine synthase	Amino acid transport and metabolism
carB	P00968	Carbamoyl phosphate synthase large chain	Nucleotide transport and metabolism
argI	P04391	Ornithine carbamoyltransferase subunit I	Amino acid transport and metabolism
gshB	P04425	Glutathione synthetase	Nucleotide transport and metabolism
tyrB	P04693	Aromatic-amino-acid aminotransferase	Amino acid transport and metabolism
pepN	P04825	Aminopeptidase N	Amino acid transport and metabolism
rbsA	P04983	Ribose import ATP-binding protein RbsA	Inorganic ion transport and metabolism
fumC	P05042	Fumarate hydratase class II	Energy production and conversion
dld	P06149	Quinone-dependent D-lactate dehydrogenase	Energy production and conversion
gor	P06715	Glutathione reductase	Energy production and conversion
melA	P06720	Alpha-galactosidase	Carbohydrate transport and metabolism
aceF	P06959	Dihydrolipoyllysine-residue acetyltransferase component of pyruvate dehydrogenase complex	Nucleotide transport and metabolism
hisB	P06987	Histidine biosynthesis bifunctional protein HisB	Amino acid transport and metabolism
hisD	P06988	Histidinol dehydrogenase	Amino acid transport and metabolism
poxB	P07003	Pyruvate dehydrogenase	Energy production and conversion
sdhB	P07014	Succinate dehydrogenase iron-sulfur subunit	Energy production and conversion
valS	P07118	Valine--tRNA ligase	Translation, ribosomal structure, and biogenesis
pheT	P07395	Phenylalanine--tRNA ligase beta subunit	Translation, ribosomal structure, and biogenesis
ilvB	P08142	Acetolactate synthase isozyme 1 large subunit	Coenzyme transport and metabolism
icd	P08200	Isocitrate dehydrogenase	Energy production and conversion
nirB	P08201	Nitrite reductase (NADH) large subunit	Energy production and conversion
ptsI	P08839	Phosphoenolpyruvate-protein phosphotransferase	Carbohydrate transport and metabolism
avtA	P09053	Valine--pyruvate aminotransferase	Amino acid transport and metabolism
hemD	P09126	Uroporphyrinogen-III synthase	Coenzyme transport and metabolism
pflB	P09373	Formate acetyltransferase 1	Energy production and conversion

putA	P09546	Bifunctional protein PutA	Transcription
gltB	P09831	Glutamate synthase [NADPH] large chain	Amino acid transport and metabolism
uvrA	P0A698	UvrABC system protein A	Replication, recombination, and repair
ackA	P0A6A3	Acetate kinase	Nucleotide transport and metabolism
ddlA	P0A6J8	D-alanine--D-alanine ligase A	Nucleotide transport and metabolism
dapA	P0A6L2	4-hydroxy-tetrahydrodipicolinate synthase	Amino acid transport and metabolism
dnaK	P0A6Y8	Chaperone protein DnaK	Posttranslational modification, protein turnover, and chaperones
htpG	P0A6Z3	Chaperone protein HtpG	Posttranslational modification, protein turnover, and chaperones
prs	P0A717	Ribose-phosphate pyrophosphokinase	Nucleotide transport and metabolism
pdxJ	P0A794	Pyridoxine 5'-phosphate synthase	Coenzyme transport and metabolism
pgk	P0A799	Phosphoglycerate kinase	Nucleotide transport and metabolism
ppa	P0A7A9	Inorganic pyrophosphatase	Energy production and conversion
rpsM	P0A7S9	Small ribosomal subunit protein uS13	Translation, ribosomal structure, and biogenesis
rpsE	P0A7W1	Small ribosomal subunit protein uS5	Translation, ribosomal structure, and biogenesis
rpoA	P0A7Z4	DNA-directed RNA polymerase subunit alpha	Transcription
tpx	P0A862	Thiol peroxidase	Posttranslational modification, protein turnover, and chaperones
talA	P0A867	Transaldolase A	Nucleotide transport and metabolism
talB	P0A870	Transaldolase B	Carbohydrate transport and metabolism
asnS	P0A8M0	Asparagine--tRNA ligase	Translation, ribosomal structure, and biogenesis
lysS	P0A8N3	Lysine--tRNA ligase	Translation, ribosomal structure, and biogenesis
lysU	P0A8N5	Lysine--tRNA ligase, heat inducible	Translation, ribosomal structure, and biogenesis
speG	P0A951	Spermidine N(1)-acetyltransferase	Translation, ribosomal structure, and biogenesis
eda	P0A955	KHG/KDPG aldolase	Carbohydrate transport and metabolism
glpC	P0A996	Anaerobic glycerol-3-phosphate dehydrogenase subunit C	Energy production and conversion
ftnA	P0A998	Bacterial non-heme ferritin	Inorganic ion transport and metabolism
ftsZ	P0A9A6	Cell division protein FtsZ	Cell cycle control, cell division, and chromosome partitioning
gapA	P0A9B2	Glyceraldehyde-3-phosphate dehydrogenase A	Nucleotide transport and metabolism
glpA	P0A9C0	Anaerobic glycerol-3-phosphate dehydrogenase subunit A	Energy production and conversion
glnA	P0A9C5	Glutamine synthetase	Nucleotide transport and metabolism
glpX	P0A9C9	Fructose-1,6-bisphosphatase 1 class 2	Carbohydrate transport and metabolism
cysB	P0A9F3	HTH-type transcriptional regulator CysB	Transcription
aceA	P0A9G6	Isocitrate lyase	Energy production and conversion
cadA	P0A9H3	Inducible lysine decarboxylase	Amino acid transport and metabolism
cfa	P0A9H7	Cyclopropane-fatty-acyl-phospholipid synthase	Cell wall/membrane/envelope biogenesis
cheZ	P0A9H9	Protein phosphatase CheZ	Translation, ribosomal structure, and biogenesis
rbsK	P0A9J6	Ribokinase	Nucleotide transport and metabolism
ybeZ	P0A9K3	PhoH-like protein	Signal transduction mechanisms

fklB	P0A9L3	FKBP-type 22 kDa peptidyl-prolyl cis-trans isomerase	Posttranslational modification, protein turnover, and chaperones
lon	P0A9M0	Lon protease	Posttranslational modification, protein turnover, and chaperones
lpdA	P0A9P0	Dihydrolipoyl dehydrogenase	Nucleotide transport and metabolism
trxB	P0A9P4	Thioredoxin reductase	Posttranslational modification, protein turnover, and chaperones
deaD	P0A9P6	ATP-dependent RNA helicase DeaD	Nucleotide transport and metabolism
adhE	P0A9Q7	Bifunctional aldehyde-alcohol dehydrogenase AdhE	Energy production and conversion
asd	P0A9Q9	Aspartate-semialdehyde dehydrogenase	Amino acid transport and metabolism
fucO	P0A9S1	Lactaldehyde reductase	Energy production and conversion
gldA	P0A9S5	Glycerol dehydrogenase	Energy production and conversion
ybiT	P0A9U3	Probable ATP-binding protein YbiT	Function unknown
ettA	P0A9W3	Energy-dependent translational throttle protein EttA	Function unknown
mreB	P0A9X4	Cell shape-determining protein MreB	Cell cycle control, cell division, and chromosome partitioning
cspC	P0A9Y6	Cold shock-like protein CspC	Transcription
ompR	P0AA16	DNA-binding dual transcriptional regulator OmpR	Transcription
rluC	P0AA39	Ribosomal large subunit pseudouridine synthase C	Translation, ribosomal structure, and biogenesis
ubiD	P0AAB4	3-octaprenyl-4-hydroxybenzoate carboxy-lyase	Coenzyme transport and metabolism
galF	P0AAB6	UTP--glucose-1-phosphate uridylyltransferase	Cell wall/membrane/envelope biogenesis
mglA	P0AAG8	Galactose/methyl galactoside import ATP-binding protein MglA	Inorganic ion transport and metabolism
hypB	P0AAN3	Hydrogenase maturation factor HypB	Transcription
kbl	P0AB77	2-amino-3-ketobutyrate coenzyme A ligase	Coenzyme transport and metabolism
ilvE	P0AB80	Branched-chain-amino-acid aminotransferase	Amino acid transport and metabolism
purB	P0AB89	Adenylosuccinate lyase	Nucleotide transport and metabolism
aroG	P0AB91	Phospho-2-dehydro-3-deoxyheptonate aldolase, Phe-sensitive	Amino acid transport and metabolism
bfr	P0ABD3	Bacterioferritin	Inorganic ion transport and metabolism
pcnB	P0ABF1	Poly(A) polymerase I	Nucleotide transport and metabolism
gltA	P0ABH7	Citrate synthase	Energy production and conversion
clpA	P0ABH9	ATP-dependent Clp protease ATP-binding subunit ClpA	Posttranslational modification, protein turnover, and chaperones
cysK	P0ABK5	Cysteine synthase A	Amino acid transport and metabolism
coaBC	P0ABQ0	Coenzyme A biosynthesis bifunctional protein CoaBC	Nucleotide transport and metabolism
menB	P0ABU0	1,4-dihydroxy-2-naphthoyl-CoA synthase	Coenzyme transport and metabolism
ychF	P0ABU2	Ribosome-binding ATPase YchF	Translation, ribosomal structure, and biogenesis
elbB	P0ABU5	Glyoxalase ElbB	Secondary metabolites biosynthesis, transport, and catabolism
aspA	P0AC38	Aspartate ammonia-lyase	Amino acid transport and metabolism
sdhA	P0AC41	Succinate dehydrogenase flavoprotein subunit	Energy production and conversion
zwf	P0AC53	Glucose-6-phosphate 1-dehydrogenase	Nucleotide transport and metabolism
glgP	P0AC86	Glycogen phosphorylase	Carbohydrate transport and metabolism

gstB	P0ACA7	Glutathione S-transferase GstB	Posttranslational modification, protein turnover, and chaperones
hemB	P0ACB2	Delta-aminolevulinic acid dehydratase	Coenzyme transport and metabolism
hns	P0ACF8	DNA-binding protein H-NS	Transcription
stpA	P0ACG1	DNA-binding protein StpA	Transcription
rob	P0ACI0	Right origin-binding protein	Transcription
lrp	P0ACJ0	Leucine-responsive regulatory protein	Transcription
crp	P0ACJ8	DNA-binding transcriptional dual regulator CRP	Transcription
allR	P0ACN4	HTH-type transcriptional repressor AllR	Transcription
gntR	P0ACP5	HTH-type transcriptional regulator GntR	Transcription
ydjA	P0ACY1	Putative NAD(P)H nitroreductase YdjA	Energy production and conversion
yeaG	P0ACY3	Uncharacterized protein YeaG	Signal transduction mechanisms
yfcZ	P0AD33	UPF0381 protein YfcZ	Function unknown
pykF	P0AD61	Pyruvate kinase I	Nucleotide transport and metabolism
guaB	P0ADG7	Inosine-5'-monophosphate dehydrogenase	Nucleotide transport and metabolism
ppnN	P0ADR8	Pyrimidine/purine nucleotide 5'-monophosphate nucleosidase	Function unknown
trmJ	P0AE01	tRNA	Translation, ribosomal structure, and biogenesis
ahpC	P0AE08	Alkyl hydroperoxide reductase C	Posttranslational modification, protein turnover, and chaperones
amn	P0AE12	AMP nucleosidase	Nucleotide transport and metabolism
corC	P0AE78	Magnesium and cobalt efflux protein CorC	Inorganic ion transport and metabolism
uvrY	P0AED5	Response regulator UvrY	Transcription
elaB	P0AEH5	Protein ElaB	Function unknown
galU	P0AEP3	UTP--glucose-1-phosphate uridylyltransferase	Cell wall/membrane/envelope biogenesis
gudD	P0AES2	Glucarate dehydratase	Cell wall/membrane/envelope biogenesis
gyrA	P0AES4	DNA gyrase subunit A	Replication, recombination, and repair
gyrB	P0AES6	DNA gyrase subunit B	Replication, recombination, and repair
minD	P0AEZ3	Septum site-determining protein MinD	Cell cycle control, cell division, and chromosome partitioning
moaB	P0AEZ9	Molybdenum cofactor biosynthesis protein B	Coenzyme transport and metabolism
nagA	P0AF18	N-acetylglucosamine-6-phosphate deacetylase	Carbohydrate transport and metabolism
nagC	P0AF20	N-acetylglucosamine repressor	Transcription
ridA	P0AF93	2-iminobutanoate/2-iminopropanoate deaminase	Translation, ribosomal structure, and biogenesis
nuoI	P0AFD6	NADH-quinone oxidoreductase subunit I	Energy production and conversion
nusA	P0AFF6	Transcription termination/antitermination protein NusA	Transcription
sucA	P0AFG3	2-oxoglutarate dehydrogenase E1 component	Nucleotide transport and metabolism
sucB	P0AFG6	Dihydrolipoyllysine-residue succinyltransferase component of 2-oxoglutarate dehydrogenase complex	Energy production and conversion
aceE	P0AFG8	Pyruvate dehydrogenase E1 component	Nucleotide transport and metabolism
parC	P0AFI2	DNA topoisomerase 4 subunit A	Replication, recombination, and repair
pmbA	P0AFK0	Metalloprotease PmbA	Function unknown
ybgI	P0AFP6	GTP cyclohydrolase 1 type 2 homolog	Function unknown
seqA	P0AFY8	Negative modulator of initiation of replication	Replication, recombination, and repair

rpe	P0AG07	Ribulose-phosphate 3-epimerase	Nucleotide transport and metabolism
relA	P0AG20	GTP pyrophosphokinase	Nucleotide transport and metabolism
spoT	P0AG24	Bifunctional (p)ppGpp synthase/hydrolase SpoT	Nucleotide transport and metabolism
rho	P0AG30	Transcription termination factor Rho	Transcription
rpsA	P0AG67	Small ribosomal subunit protein bS1	Translation, ribosomal structure, and biogenesis
serB	P0AGB0	Phosphoserine phosphatase	Amino acid transport and metabolism
sucD	P0AGE9	Succinate--CoA ligase	Energy production and conversion
tdcB	P0AGF6	L-threonine dehydratase catabolic TdcB	Amino acid transport and metabolism
yhbY	P0AGK4	RNA-binding protein YhbY	Translation, ribosomal structure, and biogenesis
rph	P0CG18	Ribonuclease PH	Translation, ribosomal structure, and biogenesis
rcsB	P0DMC7	Transcriptional regulatory protein RcsB	Transcription
bipA	P0DTT0	Large ribosomal subunit assembly factor BipA	Signal transduction mechanisms
ftsY	P10121	Signal recognition particle receptor FtsY	Cell cycle control, cell division, and chromosome partitioning
moeA	P12281	Molybdopterin molybdenumtransferase	Coenzyme transport and metabolism
udp	P12758	Uridine phosphorylase	Nucleotide transport and metabolism
glpD	P13035	Aerobic glycerol-3-phosphate dehydrogenase	Energy production and conversion
pepP	P15034	Xaa-Pro aminopeptidase	Amino acid transport and metabolism
helD	P15038	DNA helicase IV	Replication, recombination, and repair
purL	P15254	Phosphoribosylformylglycinamidine synthase	Nucleotide transport and metabolism
pepD	P15288	Cytosol non-specific dipeptidase	Amino acid transport and metabolism
malQ	P15977	4-alpha-glucanotransferase	Carbohydrate transport and metabolism
selD	P16456	Selenide, water dikinase	Nucleotide transport and metabolism
glmS	P17169	Glutamine--fructose-6-phosphate aminotransferase	Cell wall/membrane/envelope biogenesis
parE	P20083	DNA topoisomerase 4 subunit B	Replication, recombination, and repair
ycaC	P21367	Probable hydrolase YcaC	Secondary metabolites biosynthesis, transport, and catabolism
rnr	P21499	Ribonuclease R	Translation, ribosomal structure, and biogenesis
pykA	P21599	Pyruvate kinase II	Nucleotide transport and metabolism
lpxD	P21645	UDP-3-O-(3-hydroxymyristoyl)glucosamine N-acyltransferase	Cell wall/membrane/envelope biogenesis
mukE	P22524	Chromosome partition protein MukE	Cell cycle control, cell division, and chromosome partitioning
ppsA	P23538	Phosphoenolpyruvate synthase	Carbohydrate transport and metabolism
yicC	P23839	Endoribonuclease YicC	Function unknown
acnA	P25516	Aconitate hydratase A	Energy production and conversion
hflX	P25519	GTPase HflX	Translation, ribosomal structure, and biogenesis
gabD	P25526	Succinate-semialdehyde dehydrogenase	Energy production and conversion
ubiI	P25535	2-octaprenylphenol hydroxylase	Energy production and conversion
aldA	P25553	Lactaldehyde dehydrogenase	Energy production and conversion
rhlE	P25888	ATP-dependent RNA helicase RhlE	Nucleotide transport and metabolism
preA	P25889	NAD-dependent dihydropyrimidine dehydrogenase subunit PreA	Energy production and conversion
acuI	P26646	Probable acrylyl-CoA reductase AcuI	Energy production and conversion

ahr	P27250	Aldehyde reductase Ahr	Energy production and conversion
prlC	P27298	Oligopeptidase A	Amino acid transport and metabolism
tktA	P27302	Transketolase 1	Carbohydrate transport and metabolism
roxA	P27431	Ribosomal protein uL16 3-hydroxylase	Function unknown
wecE	P27833	dTDP-4-amino-4,6-dideoxygalactose transaminase	Amino acid transport and metabolism
nrdD	P28903	Anaerobic ribonucleoside-triphosphate reductase	Nucleotide transport and metabolism
treC	P28904	Trehalose-6-phosphate hydrolase	Carbohydrate transport and metabolism
ybiB	P30177	Uncharacterized protein YbiB	Amino acid transport and metabolism
sdaB	P30744	L-serine dehydratase 2	Amino acid transport and metabolism
yafC	P30864	Uncharacterized HTH-type transcriptional regulator YafC	Transcription
prpC	P31660	2-methylcitrate synthase	Energy production and conversion
nuoF	P31979	NADH-quinone oxidoreductase subunit F	Energy production and conversion
psuG	P33025	Pseudouridine-5'-phosphate glycosidase	Secondary metabolites biosynthesis, transport, and catabolism
tktB	P33570	Transketolase 2	Carbohydrate transport and metabolism
nuoC	P33599	NADH-quinone oxidoreductase subunit C/D	Energy production and conversion
nuoG	P33602	NADH-quinone oxidoreductase subunit G	Energy production and conversion
acnB	P36683	Aconitate hydratase B	Energy production and conversion
glcB	P36683	Malate synthase G	Energy production and conversion
ucpA	P37440	Oxidoreductase UcpA	Lipid transport and metabolism
aldB	P37685	Aldehyde dehydrogenase B	Energy production and conversion
rluB	P37765	Ribosomal large subunit pseudouridine synthase B	Translation, ribosomal structure, and biogenesis
uspF	P37903	Universal stress protein F	Signal transduction mechanisms
uxuB	P39160	D-mannonate oxidoreductase	Carbohydrate transport and metabolism
iadA	P39377	Isoaspartyl dipeptidase	Amino acid transport and metabolism
adhP	P39451	Alcohol dehydrogenase, propanol-preferring	Energy production and conversion
ydfG	P39831	NADP-dependent 3-hydroxy acid dehydrogenase YdfG	Function unknown
tdcG	P42630	L-serine dehydratase TdcG	Amino acid transport and metabolism
tdcE	P42632	PFL-like enzyme TdcE	Energy production and conversion
hrpA	P43329	ATP-dependent RNA helicase HrpA	Replication, recombination, and repair
uup	P43672	ATP-binding protein Uup	Function unknown
hexR	P46118	HTH-type transcriptional regulator HexR	Transcription
yhgF	P46837	Protein YhgF	Transcription
yhhX	P46853	Uncharacterized oxidoreductase YhhX	Function unknown
glcE	P52073	Glycolate oxidase subunit GlcE	Energy production and conversion
ldhA	P52643	D-lactate dehydrogenase	Energy production and conversion
can	P61517	Carbonic anhydrase 2	Inorganic ion transport and metabolism
clpB	P63284	Chaperone protein ClpB	Posttranslational modification, protein turnover, and chaperones
yheS	P63389	Probable ATP-binding protein YheS	Function unknown
yfgG	P64545	Protein YfgG	Function unknown
ygfM	P64557	Uncharacterized protein YgfM	Energy production and conversion
yqjD	P64581	Uncharacterized protein YqjD	Function unknown

ygeY	P65807	Uncharacterized protein YgeY	Amino acid transport and metabolism
ygeX	P66899	Diaminopropionate ammonia-lyase	Amino acid transport and metabolism
yhaJ	P67660	Probable HTH-type transcriptional regulator YhaJ	Transcription
fadD	P69451	Long-chain-fatty-acid--CoA ligase	Lipid transport and metabolism
gadB	P69910	Glutamate decarboxylase beta	Amino acid transport and metabolism
nrdB	P69924	Ribonucleoside-diphosphate reductase 1 subunit beta	Nucleotide transport and metabolism
ybjS	P75821	Uncharacterized protein YbjS	Cell wall/membrane/envelope biogenesis
ltaE	P75823	Low specificity L-threonine aldolase	Amino acid transport and metabolism
ybjD	P75828	Uncharacterized protein YbjD	Replication, recombination, and repair
ycbZ	P75867	Putative Lon protease homolog	Posttranslational modification, protein turnover, and chaperones
yciT	P76034	Uncharacterized HTH-type transcriptional regulator YciT	Transcription
ycjX	P76046	Ras-like GTPase YcjX	Function unknown
kdgR	P76268	HTH-type transcriptional regulator KdgR	Transcription
preT	P76440	NAD-dependent dihydropyrimidine dehydrogenase subunit PreT	Energy production and conversion
maeB	P76558	NADP-dependent malic enzyme	Energy production and conversion
hxpB	P77247	Hexitol phosphatase B	Function unknown
ykgF	P77536	Uncharacterized electron transport protein YkgF	Energy production and conversion
yajO	P77735	1-deoxyxylulose-5-phosphate synthase YajO	Energy production and conversion
oppF	P77737	Oligopeptide transport ATP-binding protein OppF	Inorganic ion transport and metabolism
xdhA	Q46799	Putative xanthine dehydrogenase molybdenum-binding subunit XdhA	Nucleotide transport and metabolism
ygeW	Q46803	Putative carbamoyltransferase YgeW	Nucleotide transport and metabolism
yqeB	Q46808	Uncharacterized protein YqeB	Posttranslational modification, protein turnover, and chaperones
ygfK	Q46811	Putative oxidoreductase YgfK	Energy production and conversion
ssnA	Q46812	Putative aminohydrolase SsnA	Nucleotide transport and metabolism
xdhD	Q46814	Probable hypoxanthine oxidase XdhD	Nucleotide transport and metabolism
bglA	Q46829	6-phospho-beta-glucosidase BglA	Carbohydrate transport and metabolism
